# Resilience measurement for environmental shocks and stressors: scale development and psychometric assessment for coastal urban informal settlements in Fiji and Indonesia

**DOI:** 10.1186/s44263-025-00177-3

**Published:** 2025-07-09

**Authors:** Hemali H. Oza, Allison P. Salinger, Ruzka Taruc, Autiko Tela, S. Fiona Barker, Karin Leder, Matthew C. Freeman, Thomas Clasen, Sheela S. Sinharoy

**Affiliations:** 1 Gangarosa Department of Environmental Health, Rollins School of Public Health, Emory University, Atlanta, GA USA; 2 Hubert Department of Global Health, Rollins School of Public Health, Emory University, Atlanta, GA USA; 3 Faculty of Public Health, Hasanuddin University, Makassar, Indonesia; 4 School of Public Health and Primary Care, College of Medicine, Nursing and Health Sciences, Suva, Fiji; 5 School of Public Health and Preventive Medicine, Monash University, Melbourne, Australia

**Keywords:** Resilience, Climate, Urban, Informal settlements, Measurement, Scale

## Abstract

**Background:**

Climate change and weather-related hazards, such as droughts and floods, pose substantial threats to the human health and well-being, especially for those in low-income households and informal settlements. Resilience, defined as the ability to cope, adapt, and recover, is critical for communities to manage these evolving threats. While there has been increased interest in ensuring that global public health and development programs contribute to resilience, the lack of valid and user-friendly resilience measurement tools limits the evidence base on the effectiveness of interventions to build resilience.

**Methods:**

We developed scales to measure economic, environmental, and social resilience to environmental shocks/stressors among urban informal settlements in low- and middle-income countries. Using an evidence-based conceptual framework, we collected data from 882 households in coastal informal settlements in Makassar, Indonesia and Suva, Fiji. We used factor analysis and item response theory approaches and assessed internal scale validity, reliability, and measurement equivalence.

**Results:**

Analysis supported a one-factor model for economic resilience, which showed a positive correlation with a financial satisfaction item, providing evidence of internal construct validity. The results also indicated a four-factor model for social resilience, with subscales for inclusion, social cohesion, collective efficacy and action, and preparedness. These subscales correlated with relevant external items—community satisfaction and perceived safety—supporting internal construct validity. The environmental resilience scale performed poorly in item response theory analysis and requires substantial refinement. The economic resilience scale demonstrated configural, metric, and scalar equivalence, suggesting that scores are comparable between households in Indonesia and Fiji. The social resilience scales showed only configural equivalence, indicating potential differences in how items relate to the underlying construct across countries. Both the economic and social resilience scales demonstrated acceptable reliability, with omega coefficients > 0.70.

**Conclusions:**

We developed and internally validated scales to measure economic and social resilience to environmental disturbances that quantify resilience as a latent construct and are grounded in resilience theory. These scales are suitable for application in urban informal settlements in Indonesia and Fiji. We recommend their use, with re-validation as needed, in the monitoring and evaluation of resilience-building interventions and policies targeting urban households in low-income settings.

**Supplementary Information:**

The online version contains supplementary material available at 10.1186/s44263-025-00177-3.

## Background

Climate change and weather-related hazards are known to adversely impact environmental determinants of human health such as air quality, water quality, food systems, and the built environment [1]. An additional 250,000 annual climate-related deaths are expected globally between 2030 and 2050 due to malnutrition, malaria, diarrhea, and heat stress—most of these deaths are predicted to occur in low- and middle-income countries (LMICs) [2]. Compounded with socio-economic challenges, climate change is likely to pose the greatest consequences to those in low-income settings, who are least likely to contribute to climate change [3, 4].

Low-income urban communities, especially those in LMICs, are vulnerable to climate change. These populations have less access to services, lower-quality and infrequently maintained infrastructure, and degraded natural environments and ecosystems as compared to urban areas in high-income communities/countries [5]. These inequalities in services, infrastructure, and environmental capital are a product of historical and systemic exploitation by the global north and further exacerbated by socio-economic marginalization and insufficient social welfare funding [6–10].

There has been an increase in urban informal settlements due to rapid and unplanned urbanization, especially in Asia and sub-Saharan Africa [11]. Generally located in more inhospitable parts of urban areas, informal settlements have no tenure security, are cut off from formal, basic services, and housing infrastructure may not comply with current planning and building regulations [12]. Urban poor communities and residents of informal settlements are vulnerable to a variety of environmental disturbances but unlike higher income settings, lack consistent and dependable resources to cope with such disturbances [5]. Global estimates from 2020 suggest over one billion people are living in either slums or other informal settlements and currently lack adequate and affordable housing. The number is expected to grow by 200 million or 20% by 2030 [13]. With a worsening climate, there is an urgent need to build resilience among these vulnerable populations [14].

Resilient households and communities are better able to cope with shocks and stressors—disruptive events or the risk of such events [15]—which leads to more efficient and timely recovery [16]. Resilience is the capacity of a system to cope with, absorb, and adapt to a changing environment and resulting disturbances, while also incorporating the ability to reorganize, learn from past experiences, and transform for future challenges [17–19]. Resilience is often considered in terms of the following capacities: “(1) absorptive capacity—the ability to minimize exposure to shocks and recover quickly when exposed; (2) adaptive capacity—the ability to make informed choices about alternative livelihood strategies based on changing conditions; and (3) transformative capacity—system-level enabling conditions for lasting resilience” [19]. There is a growing interest in building resilience among urban households and informal settlements [20]. However, there is limited evidence for resilience-building strategies, especially among urban households in LMICs and informal settlements.

A reliable and valid resilience measurement tool is needed to track progress and generate evidence towards building resilience in the face of environmental disturbances. However, resilience is a latent construct, meaning it is not directly observable or quantifiable, posing measurement challenges [21]. Existing measures often rely on direct observation, overlooking the latent nature of resilience [22]. While there is a strong theoretical foundation for resilience measurement and conceptualization, resilience frameworks require additional specificity to be applied for measures. This poses a challenge as resilience is context-specific, influenced by the system of interest (*resilience of what*), relevant shocks/stressors (*resilience to what*), and the population and cultural context (*resilience by whom*) [23]. There is little guidance on how to transform generalized frameworks into context-specific and field applicable tools/methods such as surveys and quantitative analyses. Limited resilience measurement data impedes the ability to test underlying theory and assess current programmatic approaches to building resilience [24]. Among the limited practical tools available, many have not retained the theoretical underpinnings of resilience measurement, often conflating resilience with concepts like vulnerability and mere counting of assets [25–28].

To address these challenges, we developed and validated scales measuring urban household resilience to environmental shocks/stressors in LMICs. The scales were internally validated using additionally collected data on topics such as financial satisfaction and community satisfaction and safety. The scales capture the latent construct of resilience through survey questions that assess the ability to cope, recover, and overcome relevant disturbances. This paper details the process from survey drafting to scale development and psychometric evaluation.

Our scales were developed within the ongoing Revitalizing Informal Settlements and their Environments (RISE) study, a randomized control trial across 24 informal settlements in Makassar, Indonesia and Suva, Fiji. RISE adopts a water-sensitive approach to site-specific water and sanitation infrastructure, aiming to reduce flooding hazards and fecal contamination and improve human health (see more on study design in Leder et al*.*) [29]. While not explicitly designed to improve household resilience, the participatory design approach has the potential to affect resilience through changes in social capital [30], and infrastructural interventions can affect resilience through water supply diversification and flood management [31, 32]. Resilience data produced by our study serves not only to test a previously developed theoretical resilience framework[25] but will also be used to assess the effect of the RISE program on resilience among participating informal settlements.

Bahasa Indonesia and iTaukei (Fijian) translations of the abstract are provided in Additional File 1.

## Methods

We developed three separate scales to measure economic, environmental, and social urban household resilience to environmental disturbances. We used factor analysis and item response theory (IRT) methods to evaluate the factor structure and measurement properties of the scales. Additionally, we assessed the scales’ internal validity and reliability.

### The RISE trial

Our study was conducted within RISE study, an on-going randomized control trial being conducted across 24 informal settlements in Suva, Fiji and Makassar, Indonesia with six intervention and six control settlements in each country (inception in 2017) (Additional file 1: Fig S1). Specifics of the intervention can be found in Leder et al., Francis et al., and the RISE Knowledge Product Series [29, 33–36]. Briefly, RISE uses a water-sensitive approach to participatory design of site-specific, modular, and decentralized water and sanitation infrastructural interventions. The central hypothesis of RISE is that these water-sensitive interventions will decrease environmental fecal contamination and flooding hazards, resulting overall in reduced human exposure to fecal contamination, and thus, improved human and ecological health and well-being. The infrastructural intervention includes wet pods (toilets, hand basins, rainwater tanks); pressure sewer systems; and wastewater treatment via constructed wetlands and biofilters. Stormwater management features revitalized drains, rain gardens, and permeable pavement, while flood mitigation includes backflow prevention, terrain modification, and protection walls. Water security is enhanced through municipal connections, rainwater harvesting, and well protection, with raised pathways improving road access in flood-prone areas.

### Household resilience scale development within the RISE study

To measure potential impacts of the RISE intervention on household resilience, we developed survey questions that were embedded in the September 2022 household survey (prior to the start of intervention build), which we refer to as the “main” survey throughout this paper. The rest of the methods section details our process on survey question development, data collection, scale development, further scale evaluation, and scale validation, which are in line with best practices [22, 37].

### Development of an urban household resilience framework for LMICs

For our a priori framework, we adapted the household resilience measurement framework developed in Serfillipi et al. [38]. We chose this framework as it was well grounded in resilience theory, being the result of a literature review and synthesis of 13 resilience frameworks [38]. We removed elements that would only pertain to rural or agricultural settings to create an adapted framework that would pertain to an urban environment. We further adapted the framework to make it more context-specific, based on the results of our scoping review of household and community resilience to environmental shocks and stressors in coastal urban environments in LMICs [25]. Because this framework consisted of three domains (economic, environmental, and social resilience), we chose to develop three separate resilience measurement scales, one for each of the three domains. While resilience is inherently multidimensional, with economic, environmental, and social resilience interacting to shape a household’s overall capacity to cope with disturbances, we developed separate measurement scales for each to enable independent assessment of the factors influencing each aspect of resilience.

### Survey question development

We designed the resilience module survey questions based on an adapted framework for urban household resilience measurement in LMICs, which we developed and published through a scoping review [25]. This framework guided the selection and development of survey items to capture key sub-domains, capitals, and capacities relevant to resilience in these settings. The majority of survey items were newly developed based on this framework, and five items were adapted from the regularly deployed main RISE household survey. For example, the Household Water Insecurity Experience (HWISE) scale [39], which consists of four survey questions, was already present in the main RISE survey and was therefore also used within the environmental resilience domain questions. Table S1 details the preliminary survey questions by resilience framework domain and sub-domain, indicating whether they were newly designed or adapted from the existing RISE survey. The surveys were translated into the local languages (Bahasa Indonesia for Indonesia and iTaukei for Fiji) and back-translated to English to ensure accuracy and consistency (Additional File 1: Table S1).

We carried out cognitive interviews with our new survey questions to identify and revise questions that may be unclear, ambiguous, difficult, or not understood as intended [40]. These cognitive interviews were also conducted to ensure the survey questions reflected the experiences of the current study population, and to identify potentially irrelevant questions and revise questions to better fit the context (see Additional file 1: Table S1). The goal of cognitive interviews is to reveal issues that respondents might have with survey item content or context, understanding of the items, and retrieving and integrating personal information and experiences to answer the item. Cognitive interviews are also used to reveal issues regarding sequencing, item length, sensitivity, problematic or irrelevant response options, inappropriate vocabulary, and temporal and spatial confusion [41].

We conducted six cognitive interviews with our Indonesian team members virtually via Zoom (due to COVID-19 restrictions) and nine cognitive interviews with our team members in Fiji (in person) using our first draft of survey items. During the interviews, we asked each new survey question to a staff member, recorded their response, and then followed up with additional questions to better understand how they arrived at their answer. While it is recommended to pilot survey questions with a subset of the study population, we opted to pilot our questions with staff members to minimize survey fatigue among community members. Prior to the interviews, we met with all participating in-country team members to explain the purpose of the interviews; we instructed the team members to answer the survey questions as if they were members of the RISE communities. The RISE team members that were chosen to participate in these cognitive interviews are residents of Indonesia and Fiji, and many have been working with RISE for several years. The chosen staff members have the background knowledge and cultural context of their respective countries and were able to advise on the appropriateness of the question wording, vocabulary, and translations (see Additional file 2). Survey items were revised after approximately every three interviews. After completing all the interviews, we reconvened with the RISE team members and went through each revised survey question to discuss any outstanding issues before finalizing the survey items.

### Data collection

Household surveys were conducted by in-country RISE team members from September to November 2022. The resilience module consisted of additional resilience survey questions added to the existing RISE household survey to assess economic, environmental, and social resilience. Study participants who completed the main household survey were asked if they were willing to also respond to resilience module questions, which were the focus of our scale development analysis.

The RISE trials encompass a total of 1637 households across 24 settlements in Indonesia and Fiji. For data collection, RISE staff attempted to collect data from every household within each settlement with two-revisits attempted if there was no answer on the primary contact. The survey questions were preferentially administered to (1) primary caregivers for household that contain children under 15 years, (2) female heads of households, or (3) male heads of households with no children. If the preferred respondent is unavailable, field teams make up to two additional visits. Survey respondents were compensated for their time and participation with a small gift (household items like food containers). Since the resilience module was embedded within the existing RISE household survey, it was subject to the same pre-defined preferential administration criteria [29].

### Non-response bias analysis

We performed a non-response bias analysis to compare individuals who agreed to participate in the resilience modules with those who declined. The mean age, proportion of female respondents, and median economic satisfaction level were calculated for each group. We used a two-sample *t*-test for continuous data (age), chi-squared test for binary data (gender), and a Wilcoxon rank-sum test for ordinal data (economic satisfaction). We conducted the analysis at a 95% significance level.

### Scale development analysis

Our scale development and validation approach follow guidance from public health and psychometric measurement frameworks, where validity is defined as the extent to which a scale accurately measures the intended construct and aligns with theoretical expectations. This guidance emphasizes the importance of using multiple approaches (i.e., Classical Test Theory and IRT) to assess the psychometric properties of scales and their items, rather than relying on a single method [22, 37, 40, 42–48].

To adhere to best practices for factor analysis, which require a sample size of around 600 and a complete dataset with no missing values, we removed questions with high rates of missingness (> 10%) and used pairwise delete within each domain [46]. High rates of non-response for certain survey questions may suggest poor question design due to sensitivity, irrelevance, or misunderstanding, and therefore warrant removal [37, 46]. For scale development analyses and all subsequent analyses, response options for negatively-oriented items (e.g., EC2. “How difficult would it be for your household to make ends meet after natural disasters such as a typhoons, heavy winds, storms, or floods?”) were reverse coded such that higher scores on any item, factor or scale reflect higher resilience.

The data was randomly split into two samples for EFA and CFA. Next, we used exploratory factor analysis (EFA) separately for each domain to examine internal factor structure, loadings, and model fit. EFA was necessary because while some survey questions had been previously piloted, others were either altered or newly developed, and the survey questions for each domain had not been previously used together [46]. All EFA models were run on a random split-half sample using a GEOMIN rotation and the means and variance-adjusted weighted least squares estimator, which is suitable for ordinal variables [49]. For the economic and environmental resilience domains, each containing 10 questions, we ran one to four factor EFA models. For the social resilience domain, which retained 18 questions after removing two due to high non-response rates, we ran one to six factor EFA models [49].

A high loading indicates that the observed variable is highly correlated with the underlying factor(s). Loadings range from − 1 to 1, with higher absolute values indicating a stronger relationship between the variable and the factor. Criteria for removing items from the EFA were low pattern coefficients (<|0.350|), high multidimensionality (i.e., cross-loadings (>|0.300|) on two or more factors), significant negative pattern coefficients, or poor content validity of a dimension (i.e., items loaded on factors for which only one or two items had significant pattern coefficients) [46]. When deciding whether to eliminate items, we also considered underlying construct theory to inform our decision [46, 47].

Following EFA, we then used confirmatory factor analysis (CFA) on the other random split-half sample to test the hypothesized factor structure identified in the EFA [22, 46]. We ensured that estimated loadings, or coefficients, for the identified model were large in magnitude in the expected direction (close to + 1), and that R-squared values, which measure the proportion of variance in each observed variable that is explained by the latent variables, were relatively high for each item (approximately > 0.3) [22, 46]. We assessed whether the factor structure aligned with the theoretical factors in the urban household resilience framework previously developed [25].

For all EFA and CFA models, we interpreted model fit as “good” based on the following indices and thresholds: root mean squared error approximation (RMSEA) < 0.08, comparative fit index (CFI) > 0.95, Tucker-Lewis Index (TLI) > 0.95, and standardized root mean squared residual (SRMR) < 0.08 [47]. The three CFA models for economic, environmental, and social resilience provided the factor structure for the three preliminary measurement scales that were further analyzed using IRT methods.

### IRT analysis

We utilized IRT methods after factor analysis (Classical Test Theory) to assess item-level measurement properties of retained survey questions. While CFA confirms factor structure, IRT allows us to assess how well individual items differentiated between respondents with varying levels of resilience and contributes to measurement precision. This analysis was conducted on the same sample as the CFA [22, 50]. We applied Graded Response Models (GRM)—a type of IRT model developed for polytomous data—for items with ordinal response options [48, 51]. For the binary items, 2-Parameter Logistic (2PL) models were applied and preferred over a 1PL/Rasch models as they allow for varying discrimination across items [51].

We evaluated item characteristic curves (ICCs), which illustrate the relationship between a respondent’s standing on the latent trait and the probability of choosing a specific answer choice. ICCs are used to examine how well a question is able to differentiate between respondents with low versus high levels of the trait. A binary question that differentiates between respondents well will likely produce an ICC with a distinct S-shaped curve (going from bottom left to top right for positively keyed items), with a low probability of respondents with low levels of the trait responding in the affirmative and a high probability of respondents with high levels of the trait responding in the affirmative [22]. An ordinal question that differentiates between respondents well will likely produce an ICC that shows clear separation between response options. In cases where there is no clear separation between the options, the item may not effectively measure the trait or may have problematic response options and should be considered for refinement or elimination [22].

We proceeded to evaluate the level of information or measurement precision contributed by each question relative to others within the factor. To visualize this, we produced item information curves (IICs) using the same data sample used for the CFA. An ideal IIC spans a broad range of construct levels (*x*-axis) and provides high levels of information (*y*-axis) [22]. Questions that have narrow construct ranges or low information levels were considered for refinement or elimination [22, 50].

After visually inspecting each question’s information contribution, we evaluated question discrimination and difficulty, relative to other questions within the same factor. Discrimination refers a question’s ability to distinguish between individuals with varying levels of the construct. Difficulty pertains to the range of the trait a question is able to measure (being able to measure high and low levels of the construct) [22].

We estimated discrimination and difficulty parameters for each question and used these two analyses in tandem to determine whether to retain or discard the question. We prioritized items that had higher discrimination values, relative to other questions within the same factor. We also prioritized questions that covered a unique range of difficulty (i.e., a range of difficulty not covered by other questions within the same factor). If two questions covered the same ranges, we prioritized the question with the higher discrimination level. Questions within factors with only one other question were not considered for removal since each factor must have at least two questions. We selected a 2PL model (over a 1PL model) when estimating discrimination and difficulty parameters, which considers the probability of a correct response to an item based on both the responders’ ability and the item’s discrimination and difficulty parameters[51].

### Equivalence analysis

We used multiple-group confirmatory factor analysis (on the same sample as CFA) to examine measurement equivalence for each scale across the two study populations—respondents in Makassar, Indonesia and Suva, Fiji [52]. Testing for measurement equivalence determines whether the scale questions measure the same underlying construct in both countries with comparable measurement properties between respondents in the two sites [45]. Equivalence across settings indicates that resilience scores per scale can be validly compared between settlements across Indonesia and Fiji [52].

Our analysis began with a configural equivalence model, which assesses the degree to which the factor structure of the construct being measured is comparable across settlements in Fiji and Indonesia [22]. We evaluated the fit of the configural model using the same criteria for model fit as described above for EFA and CFA. Models were estimated using a mean- and variance-adjusted weighted least squares estimation and theta parametrization [53].

Since metric equivalence is predicated on configural equivalence, we only continued with our analysis if there was evidence of configural equivalence. Metric equivalence models assess the degree to which item loadings are comparable across the two groups [22]. Similarly, scalar equivalence is predicated on metric equivalence, so we only continued our analysis if there was evidence of metric equivalence. Scalar equivalence models assess the degree to which respondents in different groups who have the same item response patterns would obtain the same factor score [22]. When examining the fit of the nested metric and scalar models, we monitored changes in RMSEA, SRMR, and CFI that corresponded to each additional restriction on model parameters, following established guidelines for assessing deteriorations in model fit statistics (∆RMSEA < 0.015, ∆SRMR < 0.030 for metric equivalence and < 0.015 for scalar equivalence, and ∆CFI > − 0.01) [45, 54, 55].

### Reliability analysis

We tested reliability to determine the internal consistency and stability of our measurement tool. We tested the reliability of each scale by calculating coefficient omega for each factor [56]. A scale is considered reliable if each factor coefficient omega is > 0.70 [56]. We used the following equation which is a function of question loadings and residual variances [41].

$$\omega=\frac{\left(\mathrm\Sigma\lambda_i\right)^2}{\left(\mathrm\Sigma\lambda_i\right)^2+\;\left(\mathrm\Sigma\theta_{\mathit{\mathcal \delta}_i}^2\right)}$$

### Convergent validity analysis

We examined convergent validity (a type of construct validity) which assesses how well a scale measures its intended construct by examining whether it behaves as hypothesized in relation to related variables based on theory, evidence, and prior research [22]. Convergent validity was assessed by examining the correlations between each scale factor and a pre-selected survey question from the regularly administered main RISE survey. These validation questions were chosen based on resilience theory and prior research, which suggested a conceptual relationship between these constructs and the resilience scale factors. We hypothesized individuals with higher economic resilience would have higher financial satisfaction [57] and individuals with higher social resilience would have higher satisfaction with their community [58, 59] and perception of safety [60]. Leveraging existing RISE survey questions allowed us to assess internal validity without introducing additional respondent burden.

### Statistical software

We used R (version 4.2.2; R Core Team, Vienna, Austria; https://www.r-project.org) within the RStudio IDE (RStudio, PBC, Boston, MA, USA) to clean the data, and Mplus (version 8.9; Muthén & Muthén, Los Angeles, CA, USA) for statistical analysis.

## Results

The household survey questions, which were developed using a conceptual framework that is described in detail elsewhere [25] and refined through a series of cognitive interviews, are presented in Table 1.

**Table 1:** Resilience module survey questions asked across 24 settlements in Makassar, Indonesia and Suva, Fiji

Question name		Survey question
Economic resilience module		
*	EC1-income	Thinking of your household’s total monthly income or weekly income, how difficult is it for your household to make ends meet, that is pay your usual expenses?
*	EC2-sustain income	How difficult would it be for your household to make ends meet after natural disasters such as typhoons, heavy winds, storms, or floods?
*	EC3-sustain income	If the main income earner in your household loses their job, how difficult would it be to make ends meet?
*	EC4-diverse income	How difficult would it be for the main income earner to find another job if they lost their current job?
	For EC5-10	Does your household have access to any of the following to help make ends meet if you need extra income?
§	EC5-savings	Access to savings
§	EC6-assets	Indonesia: Household assets (e.g., appliances, jewelry, livestock, vehicles or motorcycles) that you could sell or pawn Fiji: Household assets (e.g., appliances, jewelry, livestock) that you could sell or pawn
§	EC7-add income	Indonesia: Additional income sources (e.g., baked goods or cookies, crops, peeling and selling cashews, making and selling items, or renting out part of your house) Fiji: Additional income sources (e.g., selling eggs or food parcels, making and selling mats, baskets, fans, brooms, or renting out a part of your house)
§	EC8-transfers	Money transfers from abroad (not including loans)
§	EC9-aid	Indonesia: Aid such as good or cash from either non-profit organization, non-governmental organizations (LSM), religious or service groups, or the government Fiji: Aid such as food or cash from either non-profit organizations, non-governmental organizations, religious or service groups, or the government
§	EC10-loans	Loans
Environmental resilience module		
†	EN11-housing	In the last 4 weeks, how many times did you worry about the quality or structure of your house?
†	EN12-water^#^	In the last 4 weeks, how frequently did you or anyone in your household (including children) worry you would not have enough water for all of your household needs?
†	EN13-water^#^	In the last 4 weeks, how frequently have you or anyone in your household (including children) had to change activities or timing of activities due to problems with your water situation? Activities that may have been interrupted include caring for others, doing household chores, agricultural work, income-generating activities, sleeping, etc
†	EN14-water^#^	In the last 4 weeks, how frequently have you or anyone in your household (including children) were not able to wash hands after dirty activities (e.g., defecating or changing diapers, cleaning animal dung) because of problems with water?
†	EN15-water^#^	in the last 4 weeks, how frequently has there not been as much water to drink as you would like for you or anyone in your household (including children)?
†	EN16-electricity	In the last 4 weeks, how frequently was your household unable to afford electricity?
†	EN17-fuel	Indonesia: In the last 4 weeks, how frequently was your household unable to afford fuel for cooking such as gas? Fiji: In the last 4 weeks, how frequently was your household unable to afford fuel for cooking such as gas, kerosene, or firewood?
†	EN18-phone/internet	In the last 4 weeks, how frequently was your household unable to afford internet or phone?
†	EN19-transportation	In the last 4 weeks, how many times were you unable to access and afford your usual mode of transportation to get to where you needed to be?
†	EN20-roads	In the last 4 weeks, how many times were you unable to use the roads, access ways, or paths or were injured trying to use them? This may have been because of floods or high-water levels, obstacles in your path, or damage to the roads?
Social resilience module		
§	SO21-healthcare	Is a majority of your household able to see a doctor or healthcare worker when you or someone in your household needs medical attention?
§	SO22-shelter	Indonesia: If your household needs to evacuate because of a natural disaster, does your household has access to shelter that you feel safe going to such as public places or private places (relative’s or friend’s house)?
§	SO23-kit	Has your household prepared a disaster supply kit in case of emergencies which may include things such as cash, drinking water, food, flashlight, extra batteries, radio, whistle, local maps, etc.…?
§	SO24-leaders help	Indonesia: During natural disasters, are you and your household able to get help from RT or RW or other services? Fiji: During natural disasters, are you and your household able to get help from the police, military, community leaders, or aid groups such as the Red Cross?
§	SO25-knowledge	Do you and your household know what to do and where to go before, during, and after a natural disaster?
‡	SO26-political env	Indonesia: You and your household have access to the authorities, local government, community leaders who are in charge of the decisions that impact your day to day lives. This could include RT, RW, or Lurah Fiji: You and your household are able to communicate with community leaders, committees, advisory counselors, or elders, who are in charge of the decisions that impact your day to day lives
‡	SO27-invite	Outside of RISE related events, when there is a informal community event or gathering such as weddings, celebrations, or social gatherings, someone from your household is usually invited to attend
‡	SO28-invite	Outside of RISE events, when there is a formal community event such as a training or community meeting where decisions are discussed and made, someone from your household is usually invited
‡	SO29-roles	Some community members have roles or jobs that they do to help the community before, during, and after a natural disaster. These could include formal or informal responsibilities
‡	SO30-involved	You and your household feel involved in community decisions that will affect your household or community
‡	SO31-help	People living here are willing to help their neighbor
‡	SO32-trust	Most of the people you don’t know personally in this settlement can be trusted
‡	SO33-trust	Most of your neighbors in this settlement can be trusted
‡	SO34-trust leaders	*In less politically sensitive context (Fiji):* Most of the leaders in this settlement can be trusted *In more politically sensitive contexts (Indonesia):* When most of the leaders of this settlement make decisions/policies, the decisions are good for most households in your settlement
‡	SO35-get along	People in this settlement generally get along with each other
‡	SO36-collective efficacy	I feel confident that this community has the ability to successfully work together to overcome shocks or stressors such as natural disasters or disease outbreaks
‡	SO37-collective action	It is normal for people in this community to work together to protect from or respond to shocks or stressors like natural disasters or disease outbreak
‡	SO38-information	Your household receives information about potential or predicted natural disasters with enough time to plan and make decisions for the safety of your household
‡	SO39-information	I know where to find information on how to protect myself and my household from natural disasters
‡	SO40-plan	Planning and preparing for natural disasters is a priority for my household
Validation questions		
**	Financial satisfaction	How satisfied are you with your financial situation?
**	Community satisfaction	How satisfied are you with feeling part of your community?
**	Community safety	How satisfied are you with how safe you feel?

^*^Difficulty level response options: (1) with great difficulty; (2) with difficulty; (3) with some difficulty; (4) fairly easily; (5) easily; (6) very easily

^§^Binary response option: (0) no; (1) yes

^†^Number of times response options: (1) 0 times in the last 4 weeks (never); (2) 1–2 times in the last 4 weeks (rarely); (3) 3–10 times in the last 4 weeks (sometimes); (4) 11–20 times in the last 4 weeks (often); (5) > 20 times in the last 4 weeks (always)

^‡^Agreement level response options: (1) strongly disagree; (2) disagree; (3) agree; (4) strongly agree

^#^The 4 “water” survey questions under the environmental resilience scale were adopted from the HWISE scale [39]

^**^Scale of 0–10 (10 more satisfied)

### Descriptive statistics

Across the 24 settlements in Indonesia and Fiji, 1396 households completed the regularly administered main survey, of which 882 (63%) participated in the resilience module (Table 2).

**Table 2:** Household engagement and respondent characteristics from settlements in Indonesia and Fiji

	**Makassar, Indonesia**	**Suva, Fiji**	**Total**
Households contacted	753 (46%)	884 (54%)	1637
Households took part in main survey	593 (42%)	803 (58%)	1396
Households took part in resilience module	498 (56%)	384 (45%)	882
# of complete economic resilience subset	438 (56%)	338 (44%)	776
# of complete environmental resilience subset	487 (56%)	382 (44%)	869
# of complete social resilience subset	324 (53%)	290 (47%)	614
Average respondent age	42.3 years	44.9 years	43.8
% of Respondents that were female	91.2%	66.3%	76.9%

### Non-response bias analysis

Of the 1396 households that took part in the main RISE survey, 37% declined participation in the resilience module. Our non-response bias analysis identified statistically significant differences in the central tendency of age, gender, and economic satisfaction between respondents and non-respondents. Resilience module participants were younger (x̄_part._ = 42.0 years; x̄_non-part._ = 45.7 years), had a higher proportion of female respondents (82.4% vs 67.4%), and were significantly less satisfied with their financial status (see Additional file 1: Table S2).

### Scale development analysis

Distribution of responses to all resilience questions can be found in the supplemental section (see Additional file 1: Fig S2–Fig S6). We used exploratory factor analysis (EFA) and confirmatory factor analysis (CFA) to examine the internal factor structure of the data.

In the EFA of the economic resilience module, four items loaded onto a factor representing economic stability and two items loaded onto a factor representing financial resources, with pattern coefficients ranging from 0.77 to 0.88 for the first factor and 0.40 to 1.1 for the second factor. Fit was acceptable for the two-factor EFA model (RMSEA = 0.066, CFI = 0.974, TLI = 0.955, SRMR = 0.085). The remaining questions (EC1, EC7-EC9) were dropped as they either did not load onto any factor or cross-loaded onto multiple factors. In CFA, the standardized loadings ranged from 0.52 to 0.88 (EC2-EC5) for the first factor and 0.41 to 1.15 (EC6 and EC10) for the second factor (RMSEA = 0.118, CFI = 0.970, TLI = 0.944, SRMR = 0.068). We analyzed 10 environmental resilience module questions. In EFA, the four HWISE items (EN12-EN15) loaded onto a factor representing water insecurity and four items (EN11; EN16-EN18) loaded onto a second factor representing infrastructure and energy, with pattern coefficients ranging from 0.64 to 0.92 for the first factor and 0.40 to 0.87 for the second factor. Model fit was mixed (RMSEA = 0.156, CFI = 0.917, TLI = 0.820, SRMR = 0.043). The remaining questions (EN19-EN20) were dropped as they did not load onto any factors. In CFA, standardized loadings ranged from 0.57 to 0.91 for the first factor and 0.50 to 0.88 for the second factor. Model fit was acceptable (RMSEA = 0.088, CFI = 0.952, TLI = 0.929, SRMR = 0.057) We examined 20 social resilience module questions. We dropped questions (SO24 and SO32) due to non-response rates exceeding 10%. Based on EFA results and social resilience theory, three items (SO22, SO23, SO25) loaded on to a factor representing emergency response with pattern coefficients ranging from 0.74 to 0.87, four items (SO27–SO30) loaded onto a factor representing inclusion with pattern coefficients ranging from 0.43 to 0.62, four items (SO31, SO33–SO35) loaded onto a factor representing social cohesion with pattern coefficients ranging from 0.50 to 0.87, two items (SO36–SO37) loaded onto a factor representing collective efficacy and action with pattern coefficients ranging from 0.67 to 1.19, and three items (SO38–SO40) loaded onto a factor representing preparation and information with pattern coefficients ranging from 0.68 to 0.61. Model fit was acceptable (RMSEA = 0.059, CFI = 0.990, TLI = 0.980, SRMR = 0.063). The remaining questions (SO21, SO26) were dropped as they cross-loaded onto multiple factors. In CFA, standardized loadings ranged from 0.71 to 0.91 for the first factor, 0.62 to 0.82 for the second factor, 0.60 to 0.95 for the third factor, 0.926 to 0.929 for the fourth factor, and 0.83 to 0.95 for the fifth factor. Model fit was acceptable (RMSEA = 0.100, CFI = 0.959, TLI = 0.947, SRMR = 0.084) [43].

### IRT analysis

After identifying the factor structure, we evaluated ICCs, a form of IRT analysis which illustrates the respondents’ level of the latent trait and the probably of choosing an answer choice. ICCs for each survey item that had been retained based on the EFA and CFA results for the three scales were produced (see Additional file 1: Fig S7–Fig S11). Two of the economic questions (EC6 and EC10) were subsequently dropped since the ICCs indicated these questions did not properly distinguish between those with low and high levels of resilience. Because all other “financial resources” related questions were dropped within the economic resilience scale, we chose to drop the question asking about savings (EC5). We also dropped this question since the information provided from this item was covered in items EC2 and EC3 which asked questions related to making ends meet. Our justification was that having access to savings would contribute to a household’s ability to “make ends meet,” which is referenced in EC2.

Within the environmental resilience scale, the four “infrastructure and energy” questions (EN11; EN16-EN18) were dropped based on results of the ICCs as they showed these questions did not properly distinguish between those with low and high levels of resilience. While the four questions within the “water” factor (EN12-EN15) showed good performance in terms of ICCs, with collective evidence from EFA, CFA, and IRT, we concluded that relying solely on this factor would not be suitable for assessing overall environmental resilience. Consequently, we dropped the entire environmental scale and recommended these set of questions for significant refinement. The 16 social resilience questions performed well according to the ICCs and were all retained.

### Further IRT analysis of the social resilience scale

We examined IICs, discrimination parameters, and difficulty parameters to assess each item’s level of measurement precision and ability to distinguish between and capture varying levels of the construct, respectively. The three economic resilience questions were retained. Because of the number of items retained thus far in the economic (*n* = 3) and social (*n* = 16) resilience scales, we only continued with further IRT analysis and scale refinement with the social resilience scale.

The “emergency response” factor (SO22, SO23, SO25) provided low levels of information over a small range of the social resilience construct in comparison to other questions (Additional file 1: Fig S12), resulting in its removal from the scale. Questions “SO27-invite” and “SO33-trust” were removed due to low discrimination levels and relatively non-unique difficulty ranges (Additional file 1: Fig S13).

Table 3 summarizes all retained questions, dropped questions, the phase in which questions were dropped, and reasons for which they were dropped. Table 4 displays the factors, number of items, standardized loadings, and model fit parameters after conducting CFA again on the remaining economic and social resilience questions (finalized scales). While the RMSEA fit parameter was above recommended levels for both scales and the SRMR fit parameter was slightly above recommended levels for the economic scale, both CFI and TLI were well above the recommendation.

**Table 3:** Summary of retained and dropped items and reasons for status

Module item	Status	Phase of analysis (if dropped)	Reason
EC1-income	Dropped	EFA	Did not load on any factor
EC2-sustain income	Retained	-	Economic resilience scale in “financial stability” factor
EC3-sustain income	Retained	-	Economic resilience scale in “financial stability” factor
EC4-diverse income	Retained	-	Economic resilience scale in “financial stability” factor
EC5-savings	Dropped	IRT	Redundant item
EC6-assets	Dropped	IRT	Poor performance on ICCs
EC7-add income	Dropped	EFA	Did not load on any factor
EC8-transfers	Dropped	EFA	Did not load on any factor
EC9-aid	Dropped	EFA	Did not load on any factor
EC10-loans	Dropped	IRT	Poor performance on ICCs
EN11-housing	Dropped	IRT	Poor performance on ICCs
EN12-water	Dropped	IRT	“Water” not suitable as sole environmental resilience factor
EN13-water	Dropped	IRT	“Water” not suitable as sole environmental resilience factor
EN14-water	Dropped	IRT	“Water” not suitable as sole environmental resilience factor
EN15-water	Dropped	IRT	“Water” not suitable as sole environmental resilience factor
EN16-electricity	Dropped	IRT	Poor performance on ICCs
EN17-fuel	Dropped	IRT	Poor performance on ICCs
EN18-phone/internet	Dropped	IRT	Poor performance on ICCs
EN19-transportation	Dropped	EFA	Did not load on any factor
EN20-roads	Dropped	EFA	Did not load on any factor
SO21-healthcare	Dropped	EFA	Did not load on any factor
SO22-shelter	Dropped	IRT	Low levels of information on IICs
SO23-kit	Dropped	IRT	Low levels of information on IICs
SO24-leaders.help	Dropped	Data collection	Non-response > 10%
SO25-knowledge	Dropped	IRT	Low levels of information on IICs
SO26-political env	Dropped	EFA	Did not load on any factor
SO27-invite	Dropped	IRT	Low discrimination levels; non-unique difficulty ranges
SO28-invite	Retained	-	Social resilience scale in “inclusion” factor
SO29-roles	Retained	-	Social resilience scale in “inclusion” factor
SO30-involved	Retained	-	Social resilience scale in “inclusion” factor
SO31-help	Retained	-	Social resilience scale in “social cohesion” factor
SO32-trust	Dropped	Data collection	Non-response > 10%
SO33-trust	Dropped	IRT	Low discrimination levels; non-unique difficulty ranges
SO34-trust leaders	Retained	-	Social resilience scale in “social cohesion” factor
SO35-get along	Retained	-	Social resilience scale in “social cohesion” factor
SO36-collective efficacy	Retained	-	Social resilience scale in “collective efficacy and action” factor
SO37-collective action	Retained	-	Social resilience scale in “collective efficacy and action” factor
SO38-information	Retained	-	Social resilience scale in “preparation” factor
SO39-information	Retained	-	Social resilience scale in “preparation” factor
SO40-plan	Retained	-	Social resilience scale in “preparation” factor

**Table 4:** Scale standardized loadings and model fit statistics from CFA

Factor	Items	Std. loading	CFI	TLI	SRMR	RMSEA
Economic resilience scale			0.987	0.991	0.103^a^	0.312^a^
Financial stability	EC2-sustian.income	0.61				
	EC3-sustain.income	0.88				
	EC4-diverse.income	0.81				
Social resilience scale			0.979	0.970	0.057	0.103^a^
Inclusion	SO28-invite	0.80				
	SO29-roles	0.87				
	SO30-invovled	0.63				
Social cohesion	SO31-help	0.59				
	SO34-trust.leaders	0.76				
	SO35-get.along	1.01				
Collective efficacy and action	SO36-collective.efficacy	0.92				
	SO37-collective.action	0.93				
Preparation	SO38-information	0.97				
	SO39-information	0.92				
	SO40-plan	0.83				

^**a**^Model fit parameter is outside acceptable range (SRMR < 0.06; RMSEA < 0.08)

### Equivalence analysis across contexts

To assess whether valid comparisons of scale scores could be made across the study contexts in Indonesia and Fiji, we tested the scales for measurement equivalence (including configural, metric, and scalar) by country, using a multi-group confirmatory factor analysis (MGCFA) approach. Table 5 displays the difference in model fit statistics from configural to metric equivalence models and metric to scalar equivalence models. Configural equivalence confirms that the overall factor structure is consistent across groups, with factor loadings in the same direction; metric equivalence ensures that the relationship between items and the underlying construct is similar across groups, indicating comparable factor loadings; and scalar equivalence assesses whether the model’s thresholds are invariant, allowing for valid score comparisons across groups. The economic resilience scale demonstrated configural, metric, and scalar equivalence, indicating equivalence of the scale’s structure, loadings, and intercepts across the two contexts, indicated by the ΔCFI, ΔRMSEA, and ΔSRMR being within acceptable limits. The social resilience scale demonstrated configural equivalence, suggesting that the scale is measuring an equivalent construct across the two contexts. While there was partial evidence for metric equivalence according to the ΔSRMR, the ΔCFI and ΔRMSEA exceeded acceptable limits (Table 5). Given that ΔCFI is the main criterion for assessing equivalence [61], the metric equivalence model was rejected for the social resilience scale, indicating that the items may have a varying relationship to the underlying construct being measured in each country. Having rejected the metric model, we also rejected the scalar equivalence model for the social resilience scale. Table S3 displays the model fit statistics for the configural, metric, and scalar models for the economic and social resilience scales (Additional file 1: Table S3).

**Table 5:** Differences in model fit parameters to establish measurement equivalence across groups by scale

Difference in model fit parameters (recommended limit)	Economic resilience scale		Social resilience scale	
	**Configural a metric**	**Metric a scalar**	**Configural a metric**	**Metric a** **scalar**
ΔCFI (> − 0.01)	0.001	− 0.003	− 0.035^a^	0.034
ΔRMSEA(< 0.015)	− 0.036	0.009	0.077^a^	− 0.077
ΔSRMR (metric: < 0.030; scalar: < 0.015)	− 0.018	− 0.009	0.020	− 0.012

^a^Model fit parameter is outside acceptable range indicating potential misfit of model to the data

### Reliability

We tested reliability to determine the internal consistency of our measurement tool. Both the economic and social resilience scales showed acceptable levels of reliability with coefficient omegas ranging from 0.78 to 0.93. Coefficient omega scores are generally considered acceptable when > 0.70 [56]. Table 6 displays coefficient omegas per factor per scale.

**Table 6:** Reliability analysis through coefficient omega values by subconstruct and construct validity analysis through correlations

Scale	Factor	Reliability	Convergent validity	
		**Omega coefficient**	**Validating variable**	**Correlation**
Economic resilience	Financial stability	0.81	Financial satisfaction	0.464*
Social resilience	Inclusion	0.81	Community satisfaction	0.296*
	Social cohesion	0.84	Community satisfaction	0.252*
	Collective efficacy and action	0.93	Community satisfaction	0.420*
	Preparation	0.87	Safety	0.232*

^*^Correlation is statistically significant at a 99% confidence level (*p*-value < 0.01)

### Validity

We examined internal convergent validity of the scales to examine the adequacy of each scale to measure the intended construct. Table 6 displays the correlations between factors among the economic and social resilience scales and the variables used to validate the factors. The positive correlation direction and significance at a 99% confidence level support the factors having construct validity.

## Discussion

We developed and internally validated scales to measure economic and social resilience, while recommending significant refinement of the environmental resilience scale. The economic and social resilience scales presented in this study are novel measurement tools that (1) quantify resilience as a latent construct, (2) are conceptually grounded in resilience theory, and (3) are contextualized for urban households to measure resilience to environmental disturbances. These scales are validated for use within urban informal settlements in Fiji and Indonesia, a population often overlooked and understudied in resilience research [25, 62]. The economic and social scales demonstrate clear reliability and dimensionality, based on factor analysis. Our IRT analysis further revealed that retained questions demonstrated measurement precision. The scale questions differentiate between varying levels of economic and social resilience, while also collectively capturing a diverse range of trait ability.

Our data supported configural equivalence for the revised economic and social resilience scales, regarding measurement equivalence across country settings (Indonesia and Fiji). This suggests that the factor structure of the scales measured salient constructs of economic and social resilience consistently across the two settings. Metric equivalence was supported for the economic scale, indicating that the scale questions were interpreted similarly across the two settings. Scalar equivalence was also supported for the economic resilience scale, indicating that the scores can be compared between Indonesia and Fiji. Scalar equivalence is crucial for making valid and meaningful score comparisons between groups [22, 52]. The scales are meaningful representations of the concepts they endeavor to measure in each context but these concepts function somewhat differently depending on context.

To calculate scores, we recommend using factor loadings from the CFA as weights for each item by factor. This approach provides more accurate scores than traditional sum scoring, which assumes that all items have the same relationship to the factor [22]. Scores can be calculated by summing weighted item scores within each factor. Because the social and economic resilience scales were developed and analyzed independently, they can be administered and scored independently. Because this study internally validated the scales for use in Fiji and Indonesia, re-validation is required to adapt these measures for other settings. The framework used to develop the scales was designed to capture the multidimensional nature of resilience, including its economic, environmental, and social domains. Similar studies that assessed urban resilience in LMICs have also utilized these three domains [63–68]. Some have also used domains such as governance [63], institutions [64–67], and infrastructure [68], potentially due to their focus on community resilience, as opposed to household resilience.

Our scales diverge from prior studies in two main ways. First, our study incorporates absorptive and adaptive capacities rather than focusing on household capitals alone, providing a more dynamic and action-oriented perspective [62, 69, 70]. The economic resilience questions assess absorptive and adaptive capacities by measuring a household’s ability to cope with and recover from financial disruptions. Absorptive capacity is reflected in how well a household can withstand immediate economic shocks, such as job loss or disaster-related income disruption, without experiencing severe financial strain. Adaptive capacity is demonstrated in a household’s ability to adjust to changing conditions, such as seeking new employment or adopting alternative income-generating strategies to sustain financial well-being over time.

The social resilience questions capture absorptive and adaptive capacities by assessing community engagement, trust, collective action, and access to information. Absorptive capacity is reflected in a household’s ability to leverage social networks and community support to withstand immediate shocks, such as receiving timely disaster information, participating in preparedness activities, and relying on trusted leadership. Adaptive capacity is demonstrated through active participation in community decision-making, collaboration in disaster response efforts, and access to training or leadership roles, which enable households to adjust strategies and strengthen resilience over time. Together, these elements enhance a community’s ability to collectively prepare for, respond to, and recover from environmental and socio-economic disruptions.

This is a different approach than many previous studies that have used indices or sets of indicators to assess resilience, which are generally used to assess observable phenomena [63, 65–69, 71, 72]. Third, unlike many previous studies that focus on one singular shock or stressor [63, 64, 68], we designed our scales to capture resilience to multiple environmental disturbances relevant to coastal urban informal settlements in Fiji and Indonesia (floods, cyclones/typhoons, heavy winds, storms, natural disasters where relevant) rather than anchoring responses to a single hazard type (e.g., just flooding). This approach ensures that the scales reflect resilience in the context of the most prevalent and impactful environmental threats in these settings. While traditional resilience approaches emphasize resilience to a specific shock/stressor (*resilience to what*). We sought to encompass a range of shocks and stressors to which the system and population of interest are vulnerable. This approach recognizes that human systems—and disturbances—do not exist in isolation, and real-world contexts involve multiple vulnerabilities to various and concurrent [73].

Despite the abundance of studies assessing urban resilience in Indonesia [63, 64, 69, 71, 74], there is a notable gap in the evidence regarding resilience in Fiji. Our study begins to address this gap, recognizing that both countries are home to numerous informal settlements and coastal communities vulnerable to the impacts of climate change, including sea level rise, erratic precipitation patters, flooding, and the heightened frequency and intensity of storms and cyclones in the region. The validation of scales in these diverse contexts strengthens the understanding of resilience dynamics in the face of climate change-induced challenges.

The scales provide a reliable and valid means of measuring household resilience in urban informal settlements in Fiji and Indonesia—a population frequently overlooked in resilience research [62, 75–77]. While evidence remains relatively scarce, a few studies have assessed resilience within informal settlements. However, these studies only focused on the physical quality of settlements as a crucial component of adaptive capacity in informal settlements in Indonesia [69]. Gaisie et al. highlighted the role of social capital in resilience outcomes within Ghanaian informal settlements which was found to motivate preparedness and mitigate impacts and facilitate recovery from flooding, emphasizing the significance of incorporating social domains in resilience measurement [62]. Additional studies, while not exclusively focused on informal settlements, have recognized them as vulnerable communities with specific needs and distinct pathways toward resilience [70, 78].

Our environmental resilience scale did not adequately differentiate between respondents with low versus high levels of the trait and is not recommended for use. The scale will be further refined to cover initially dropped sub-domains and improve wording of the questions to better capture system capacities.

Due to limitations in the number of new survey questions that could be added to the survey, we had to prioritize certain sub-domains and consequently dropped five of them, including ecological services, sanitation, and food, which were part of the environmental resilience domain. Moving forward, we aim to develop and evaluate survey questions that encompass these sub-domains.

This poor performance may be attributed to the lack of framing and connection to absorptive or adaptive capacities in relation to the occurrence of disturbances. Unlike the economic and social resilience questions, which were often asked in the context of natural disasters, the environmental resilience questions solely focused on access to services/utilities and infrastructure. For example, EC2-sustain.income asks “How difficult would it be for your household to make ends meet after natural disasters such as [example]?” which is asking about the ability to absorb financial difficulty in the face of an environmental disturbance. In contrast, for example, EN11-housing asks “In the last 4 weeks, how many times did you worry about the quality or structure of your house?” which does not reference the household facing a disturbance. To refine this question, we may instead ask something like “Do you worry about the quality and structure of your house during a natural disaster such as [example]?” or “Would your household be able to maintain and repair the structure of your house after a natural disaster such as [example]?” To refine this aspect, we suggest modifying the questions to explicitly reference disturbances, such as natural disasters, to capture the capacities of households to maintain and repair the structure of their houses in such events.

This study had several limitations that may have affected the validity of the scales. First, over a third of approached households declined to participate in the resilience module, leading to potential non-response bias. Statistical differences were observed for age, gender, and economic satisfaction between participants and non-participants. The sample may not fully represent the diversity of the target population, potentially leading to an under or overrepresentation of certain socio-economic statuses, cultural groups, ages, or genders. These biases should be considered when interpreting and generalizing the findings.

Second, due to resource and logistical constraints, we were unable to conduct cognitive interviews with informal settlement community members. Instead, we opted to interview in-country staff who have knowledge of the setting, communities, and language. While cognitive interviews are ideally conducted within the study population, testing our modules with in-country staff, who are intimately familiar with the communities and culture, may strengthen the validity of our scales. Third, the length of the survey constrained the number of survey items that could be tested, and we had to prioritize certain items over others. This may have impacted the content validity of the scales, as some important dimensions of resilience may not have been fully captured. Fourth, while the social resilience scale demonstrated configural equivalence, the metric and scalar models were rejected based on MGCFA analyses. Therefore, comparisons of the magnitude of results should be made with caution. Despite these limitations, the scale demonstrated clear dimensionality and strong psychometric properties, reliability, and internal convergent validity. Lastly, our item development process did not incorporate inductive methods, such as qualitative interviews, to identify domains and sub-domains. However, we relied on resilience theory, prior research, an adapted framework for urban LMIC populations, and cognitive interviews with research staff for item refinement and to ensure alignment with lived resilience experiences.

## Conclusions

The data collection, analysis, and results presented in this study contribute to addressing the gap in resilience data, essential for testing resilience theory and generating evidence. Going forward, the scales will play a crucial role in evaluating the effect of the RISE interventions on household resilience. We anticipate that our scales will prove a useful tool, enabling practitioners to identify areas of low resilience and to design interventions and programs that build resilience in these vulnerable communities, coupled with the ability to monitor progress.

## Supplementary Information

Additional file 1. Native language abstracts in Bahasa Indonesia and iTaukei (Fijian), supplementary information on survey design and supplementary results of factor analysis, item response theory analysis, and measurement equivalence.

Additional file 2. Cognitive interview guide.

## Data Availability

Answers to survey questions are not publicly available to maintain privacy of the participants. They may be made available from the corresponding author on reasonable request.
