# Supplementary material for: Resilience measurement for environmental shocks and stressors: scale development and psychometric assessment for coastal urban informal settlements in Fiji and Indonesia
Source: BMC Glob Public Health. 2025 Jul 9;3:61. doi: 10.1186/s44263-025-00177-3 (PMC12243337; doi:10.1186/s44263-025-00177-3)
Supplement: Supplementary file 1 — Additional file 1. Native language abstracts in Bahasa Indonesia and iTaukei (Fijian), supplementary information on survey design and supplementary results of factor analysis, item response theory analysis, and measurement equivalence. [file 44263_2025_177_MOESM1_ESM.docx]

**Additional File 1**

Publisher’s note: **This translation in Bahasa Indonesia was submitted by the authors and we reproduce it as supplied. It has not been peer reviewed. Our editorial processes have only been applied to the original abstract in English, which should serve as reference for this article. This translated abstract is published under the same license as the article.**

**Bahasa Indonesia Native Language Abstract**

**Terjemahan**

**Pendahuluan:** Dampak bencana alam yang diakibatkan oleh perubahan iklim dan cuaca, seperti kekeringan dan banjir, merupakan ancaman besar bagi kesehatan dan kesejahteraan manusia, utamanya bagi keluarga yang berpenghasilan rendah dan tinggal di wilayah permukiman informal. Resiliensi, didefinisikan sebagai kemampuan untuk mengelola, beradaptasi, dan pulih dari bencana, merupakan hal yang sangat penting bagi masyarakat dalam menghadapi bencana yang semakin meningkat. Meskipun ada peningkatan kepedulian dalam memastikan bahwa program pengembangan dan kesehatan masyarakat global berkontribusi pada resiliensi, kurangnya alat ukur resiliensi yang valid dan mudah diaplikasikan membatasi basis data terkait efektivitas intervensi dalam membangun ketahanan masyarakat.

**Metode:** Kami mengembangkan skala untuk mengukur ketahanan ekonomi, lingkungan, dan sosial terhadap guncangan/tekanan lingkungan pada permukiman informal daerah perkotaan di beberapa negara berpenghasilan rendah dan menengah. Dengan menggunakan kerangka konseptual berbasis data-empiris, kami mengumpulkan data dari 882 keluarga di wilayah permukiman informal di daerah pesisir kota Makassar, Indonesia, dan Suva, Fiji. Kami menggunakan metodologi pendekatan analisis faktor, Teori Respons Butir (IRT) serta menilai validitas skala internal, reliabilitas, dan invarian pengukuran.

**Hasil:** Analisis ini mendukung penerapan model satu faktor untuk ketahanan ekonomi, yang menunjukkan korelasi positif dengan indikator kepuasan finansial, dengan memberikan dukungan empiris terhadap validitas konstruk internal skala tersebut. Hasil juga mengindikasikan empat faktor model untuk resiliensi sosial, yang terdiri dari subskala inklusi, kohesi sosial, efikasi dan upaya kolektif, serta kesiapsiagaan. Keempat subskala ini menunjukkan korelasi yang signifikan dengan indikator eksternal yang relevan yakni kepuasan masyarakat dan persepsi mereka terhadap keamanan yang turut memperkuat validitas konstruk internalnya. Skala ketahanan lingkungan menunjukkan performa yang buruk dalam analisis teori respons butir (item response theory) dan memerlukan perbaikan dan pengembangan lanjutan untuk mencapai tingkat validitas pengukuran yang memadai. Skala ketahanan ekonomi menunjukkan kesetaraan konfigural, metrik, dan skalar, yang mengindikasikan bahwa skor yang diperoleh dapat dibandingkan secara valid antar rumah tangga di Indonesia dan Fiji. Skala ketahanan sosial hanya memenuhi kesetaraan konfigural, yang mengindikasikan adanya perbedaan dalam hubungan antara item pengukuran dengan konstruk dasar di masing-masing negara. Meskipun demikian, baik skala ketahanan ekonomi maupun sosial menunjukkan tingkat reliabilitas yang dapat diterima, dengan koefisien omega lebih dari 0,70.

**Kesimpulan:** Kami telah mengembangkan dan melakukan validasi internal instrumen skala untuk mengukur resiliensi ekonomi dan sosial terhadap tekanan lingkungan, yang mengkuantifikasi resiliensi sebagai suatu konstruk laten dan berlandaskan pada teori resiliensi. Skala-skala ini dinilai sesuai untuk diterapkan di permukiman informal perkotaan di Indonesia dan Fiji. Kami menyarankan penggunaan skala ini, dengan validasi ulang sesuai kebutuhan, dalam kerangka pemantauan dan evaluasi intervensi serta kebijakan pembangunan resiliensi yang ditujukan bagi rumah tangga perkotaan di wilayah berpendapatan rendah.

Publisher’s note: **This translation in Fijian was submitted by the authors and we reproduce it as supplied. It has not been peer reviewed. Our editorial processes have only been applied to the original abstract in English, which should serve as reference for this article. This translated abstract is published under the same license as the article.**

**iTaukei (Fijian) Native Language Abstract**

**Tukutuku Taumada:** Na draki veisau kei na vei draki drakidrakita eso me vaka na dravuisiga kei na waluvu, e salavata mai na veibolebole, ka bolei kina na bula ni dua na tamata, qo ena veitiki kece sara ni nona bula, ka vakauasivi vei ira na vuvale ka ra sega soti ni rawa ka vinaka sara, ka vakatalega kina vei ira ka ra vakaitikotiko e nai tikotiko e sega ni vakadeitaki vakamatanitu. Na yaloqaqa se yalo bolebole e cavuti vei ira na so ka ra rawa ni bolea na dredre, bula kaya na dredre koya, ka rawa talega ni lako tani mai na dredre ni bula ka ra lako curuma voli , ka sa dodonu kina ena veitikotiko eso mera cakacakataki ira ena veidredre oqori.E dina ga ni sa tubu tiko na nodra kauwai ena noda vuravura me baleta na bula raraba kei na veituvatuva eso ka sa qaravi tiko mai, e se tiko ga na luluqa ni veiqaravi ena kena railesuvi ni veituvatuva eso, ka sagai tiko kina na toso ki liu ena kena rawati na yaloqaqa kei na yalo bolebole.

**Tuvatuva eso me qaravi:** E a mai vakayacori e dua na vakadidike me laurai kina na kena vakaveiwekanitaki na bula ni rawaka vakailavo, bula ni veiwekani vakamataitikotiko kei na drakidrakita ni bula ka sotavi mai na draki veisau eso, oqo e vakabibitaki vei ira na vakaitikotiko ena veivanua eso ka sega ni tudei. E na kena a vakayacori e dua na vakadidike me vakaoqo, keimami a vakasokumuna mai eso na tukutuku mai na 882 na vuvale ena veitikotiko sega ni tudei volekata na baravi mai Makassar, Indonesia kei Viti e Suva. E vakayagataki eke e rua na gaunisala ni vakasokumuni tukutuku me rawati kina na usutu ni ka ga gadrevi sai koya na “factor analysis” kei na “item response theory” ka varautaki me rawati kina e dua na itukutuku ka vakabauti.

**Veika e rawati:** Na vakadidike e tokona na one-factor model me baleta na vorati na leqa ni bula vakailavo, e vakaraitaki ni veiwekani Vinaka kei na marautaki ni veika vakailavo, e vakaraitaki ni vakadinadina ni internal construct validity. Na veika e rawati e vakaraitaka e Va na Factor model ni kena vorati na leqa na bula ni vakaveiwekani. Na i vakarau ni nomuni vakaitavi, duavata vakaveiwekani,yalovata na kena sagai me rawati na cakacaka vata, kei na bula vakavakarau. Na i vakarau ni veiwekani e veiganiti kei na veika e so e oka kei na bula marau vakamata-itikotiko kei na nodra rai me baleta na nodra taqomaki, e tokona na internal construct validity. Na vakarau e vakayagataki me dikevi kina na leqa ni yau bula e dravudravua na kena vakasokomunitaki mai nai tukutuku, e gadrevi me vakavoutaki. Na vakarau e vakayagataki e na kena sokomuni na I tukutuku ni vorati ni leqa ni bula vakailavo e vakaraitaka ni veiganiti, vakatautauvataki na macala ni vakadidike e rawati e na vei dui vuvale e viti kei Indonesia. Na vorati na leqa ni bula vakaveiwekani e vakaraitaka na configural equivalence, ka vakaraitaka na duidui levu ni veika e ra lako curuma e na nodra dui matanitu. Na I vakarau e vakayagataki e na kena vakasokomuni na I tukutuku ni rawa ka vakailavo kei na vorati ni leqa ni bula vakaveiwekani e vakadonui ka ciqomi, vata kei na omega coefficiens > 0.70.

**Itinitini:** E buli ka vakadonui e dua na i vakarau me vakayagataki ena kena vakarautaki na rawa ka vakailavo kei na vorati ni leqa vakaveiwekani kina veiveisau e yaco kina vei yaubula ka vuna na kena gadrevi me vakarautaki na leqa e ra lako curuma ka volai e na vola itukutuku ni resilience theory. Na i vakarau e veiganiti e na kena vaka yagataki e na veitikotiko e loma ni tauni e sega ni vakadeitaki vakamatanitu e Indonesia kei Viti. E vaka tututaki na vaka yagataki kei na vaka donuitaki nai I vakarau e gadrevi e na kena vakaraici kei na kena dikevi ni resilience-building interventions kei na veilawa e so vaka tabakidua vei ira na vuvale sega ni rawa ka vakailavo vinaka e loma ni tauni.

**Table S1: Original and revised survey questions by resilience domains and sub-domains with justifications for revision or removal**

* During the cognitive interviews, the in-country staff members recommended using the term 'disasters' instead of specifying particular hazards in several survey questions. This choice of terminology was made to enhance the effectiveness and relatability of the survey for community members responding to it.

| **Domain** | **Sub-domain** | **Pre-existing vs. newly designed questions** | **Original Question** | **Reason for revision** | **Finalized questions*** |
| --- | --- | --- | --- | --- | --- |
| **Economic Resilience** | **Livelihood (Stability)** | Pre-existing | Thinking of your household's total monthly income or weekly income, how difficult is it for your household to make ends meet, that is pay your usual expenses? | No revision | EC1 Thinking of your household's total monthly income or weekly income, how difficult is it for your household to make ends meet, that is pay your usual expenses? |
|  |  | New | How difficult is it for your household to make ends meet after an environmental shocks and stressors such as a typhoon, flood, or disease outbreak? | Revised – Clearer wording | EC2 How difficult would it be for your household to make ends meet after natural disasters such as [example]? |
|  |  | New | If someone in your household loses their job, how difficult is it to make ends meet? | Revised – Added ‘main income earner’ to standardize who respondent thinks of losing their job | EC3 If the main income earner in your household loses their job, how difficult would it be to make ends meet? |
|  |  | New | How difficult is it for the main income earner to find another job if they lost their current job? | Revised – Added ‘main income earner’ to standardize who respondent thinks of losing their job and having to find a new one | EC4 How difficult would it be for the main income earner to find another job if they lost their current job? |
|  | **Financial Resources (Stabilizing)** | New | During or after environmental shocks and stressors such as typhoons, floods, droughts, or disease outbreaks, does your household have access to **savings** that could be used to help support you and your household? | Revised – Simplified into one question with the responder prompted to indicate all that apply. We also added questions to ask about additional income sources and loans. | EC5 Does your household have access to any of the following to help make ends meet if you need extra income?  1. Access to savings  2. Household assets (e.g., [example]) that you could sell or pawn  3. Additional income sources (e.g., [examples])  4. Money transfers from abroad (not including loans)  5. Aid such as good or cash from either non-profit organization, non-governmental organizations ([example]), religious or service groups or the government  6. Loans |
|  |  | New | Does your household have **assets** that could be sold for additional income if needed? This may include household assets or other assets such a livestock. |  |  |
|  |  | New | If your household is need of additional income, do you or someone in your household have access to **money** **transfers** from outside your community or abroad? |  |  |
|  |  | New | After environmental shocks or stressors, do you or someone in your household (including children) receive any **aid** or assistance such as food, cash, or other support from anyone? This could include local, regional, or national government, non-profits, religious organizations, non-governmental organizations, or other service groups. |  |  |
| **Environmental Resilience** | **Ecological services** | NA | No question asked | NA | NA |
|  | **Energy/**  **electricity** | New | In the last 4 weeks, how frequently did you or anyone in your household worry that your main source of electricity, internet, phone, and gas may stop working? | Revised – The original question was separated out to only ask about one utility at a time. Original question was also separated into EN18 (see ICT sub-domain). | EN16 In the last 4 weeks, how frequently was your household unable to afford electricity?  EN17 In the last 4 weeks, how frequently was your household unable to afford fuel such as [examples of fuel]? |
|  | **Housing and Shelter** | New | In the last 4 weeks, how many times did you worry about the quality or structural integrity of your house? |  | EN11 In the last 4 weeks, how many times did you worry about the quality or structure of your house? |
|  | **Information and communication technology (ICT)** | New | *See question for ‘Energy/electricity’ sub-domain* | Revised – *See question for ‘Energy/electricity’ sub-domain* | EN18 In the last 4 weeks, how frequently was your household unable to afford internet or phone? |
|  | **Land use** | NA | No question asked | NA | NA |
|  | **Sanitation** | NA | No question asked | NA | NA |
|  | **Transportation** | New | In the last 4 weeks, how many times were you unable to access transportation to get to somewhere you needed to be? | Revised – We added ‘afford’ as we got feedback that sometimes transportation is prohibited by affordability as well. | EN19 In the last 4 weeks, how many times were you unable to access and afford your usual mode of transportation to get to where you needed to be? |
|  |  |  | In the last 4 weeks, how many times were you concerned that you would not be able to use the roads and/or accessways to get to where you needed to be? This may have been because of floods or high-water levels, obstacles in your path, or damage to the road that made it unusable. | Revised – We added the aspect of being injured when trying to use roads and access ways because we received feedback that even though roads may be flooded or have obstacles, many people still use them but are injured in the process. | EN20 In the last 4 weeks, how many times were you unable to use the roads, access ways, or paths or were injured trying to use them? This may have been because of floods or high-water levels, obstacles in your path, or damage to the roads? |
|  | **Water** | Pre-existing | HWISE_WORRY: In the last 4 weeks, how frequently did you or anyone in your household (including children) worry you would not have enough water for all of your household needs? | No revision | EN12: HWISE_WORRY: In the last 4 weeks, how frequently did you or anyone in your household (including children) worry you would not have enough water for all of your household needs? |
|  |  | Pre-existing | HWISE_PLANS: In the last 4 weeks, how frequently have you or anyone in your household (including children) had to change activities or timing of activities due to problems with your water situation? | No revision | EN13 HWISE_PLANS: In the last 4 weeks, how frequently have you or anyone in your household (including children) had to change activities or timing of activities due to problems with your water situation? |
|  |  | Pre-existing | HWISE_HANDS: In the last 4 weeks, how frequently have you or anyone in your household (*including children) were not able to wash hands after dirty activities (e.g., defecating or changing diapers, cleaning animal dung) because of problems with water? | No revision | EN14 HWISE_HANDS: In the last 4 weeks, how frequently have you or anyone in your household (*including children) were not able to wash hands after dirty activities (e.g., defecating or changing diapers, cleaning animal dung) because of problems with water? |
|  |  | Pre-existing | HWISE_DRINK: in the last 4 weeks, how frequently has there not been as much water to drink as you would like for you or anyone in your household (including children)? | No revision | EN15 HWISE_DRINK: in the last 4 weeks, how frequently has there not been as much water to drink as you would like for you or anyone in your household (including children)? |
| **Social Resilience** | **Community & institutional environments** | New |  | Added – We added this new question because we received feedback that community authorities and local leaders are crucial in coping with disturbances. | SO24 During natural disasters, are you and your household able to get help from [example]? |
|  |  | New | Do you agree or disagree: When most of the leaders of this settlement make decisions/policies, they are accepted and good for most households in your settlement. | Revised – We received feedback that while, this is a culturally appropriate way to ask the question in Indonesia where the political culture is more sensitive, we can ask in a more straightforward manner in Fiji. | SO34 ***Depending on level of cultural sensitivity*** *In less politically sensitive context (Fiji):* Most of the leaders in this settlement can be trusted.  *In more politically sensitive contexts (Indonesia):* When most of the leaders of this settlement make decisions/policies, the decisions are good for most households in your settlement. |
|  |  | New | Do you agree or disagree: You and your household have access to the authorities, local government, community leaders who are in charge of the decisions that impact your day to day lives. | Revised – We changed the wording of “access to” to “communicate with” for clarity | SO26 You and your household are able to communicate with [example] who are in charge of the decisions that impact your day to day lives. [This could include [example]]. |
|  |  | New | Do you agree or disagree: When there is a community events, gatherings, trainings, or meetings, someone from my household is usually invited to attend. | Revised – We separated this question to ask about formal and informal events separately, since many of the settlement events are related to RISE. | SO27 Outside of RISE related events, when there is a [[informal/formal]] community event or gathering such as [example], someone from your household is usually invited to attend.  SO28 Outside of RISE events, when there is a formal community event such as a training or community meeting where decisions are discussed and made, someone from your household is usually invited. |
|  |  | New | Do you agree or disagree: Some community members have pre-defined responsibilities or jobs that they do to help the community when there is a disaster, shock, stressor or emergency. | Revised the wording for clarity. We received feedback that some of the roles can be formally bestowed onto a community member or may be an informal role. We revised the question to reflect this feedback. | SO29 Some community members have roles or jobs that they do to help the community before, during, and after a natural disaster. These could include formal or informal responsibilities. |
|  |  | New | Do you agree or disagree: You and your household feel involved in community decisions that will affect your household, cluster, or community. | Revised – We removed the term ‘cluster’ as the meaning of the term was unclear and confusing. | SO30 You and your household feel involved in community decisions that will affect your household or community. |
|  |  | Pre-existing | Do you agree or disagree: people living here are willing to help their neighbor | No revision | SO31 People living here are willing to help their neighbor. |
|  |  | New | Do you agree or disagree: most people living in this settlement can be trusted | Revised – We received feedback that whether or not someone knows the person in the community may change if they trust them. We split the question to ask about people respondents know personally and those they may not know personally. | SO32 Most of the people you don't know personally in this settlement can be trusted.  SO33 Most of your neighbors in this settlement can be trusted. |
|  |  | Pre-  existing | Do you agree or disagree:  People in this community generally do not get along with each other | Revised – This question was originally negatively worded, meaning the question was inversely related to social resilience. We removed ‘do not’ to make sure the question would be directly related to the construct. | SO35 People in this settlement generally get along with each other. |
|  |  | New | Do you agree or disagree: People in this community would contribute money or labor to households that needed repairs after a disaster even if they were not family. | Removed – We received feedback that respondents would likely only answer strongly disagree as they very rarely had funds to spare and would not be willing to spare extra funds in this manner. |  |
|  |  | New | I feel confident in my ability to contribute to the community's efforts to overcome shocks/stressors | Removed – We received feedback that this was similar to question SO36. |  |
|  |  | New | I feel confident that this community has the ability to successfully work together to overcome shocks/stressors | No revision | SO36 I feel confident that this community has the ability to successfully work together to overcome shocks or stressors such as natural disasters or disease outbreaks. |
|  |  | New | I can influence decisions about how the community will protect itself from or respond to shocks/stressors | Removed – similar to SO30 |  |
|  |  | New | Anyone in the community who wants to can be involved in decision-making about how the community will protect itself from or respond to shocks/stressors | Removed – we received feedback that this question was confusing in terms of what “who wants to can be involved.” We felt that SO30 covered the information we were seeking. |  |
|  |  | New | I or members of my household usually contribute to community efforts to protect from or respond to shocks/stressors | Removed |  |
|  |  | New | It is normal for people in this community to work together (meaning NOT every household for themselves) to protect from or respond to shocks/stressors. | Revised – Revised to reduce unnecessary and unclear wording. | SO37 It is normal for people in this community to work together to protect from or respond to shocks or stressors like natural disasters or disease outbreak. |
|  |  | New | People in this community are motivated to overcome shocks/stressors, even when it seems challenging to do so | Removed – similar to question SO37 |  |
|  |  | New | Working together/doing communal work to protect the community from shocks/stressors before they happen is a good use of our time and/or money. This might include giving money, labor, or land for digging drainage ditches or building sea walls. | Removed – similar to question SO37 |  |
|  |  | New | Working together/doing communal work to clean up or rebuild the community after shocks/stressors is a good use of our time and/or money. This might include community clean ups to clear debris or giving money or labor to rebuild communal structures. | Removed – similar to question SO37 |  |
|  | **Education** | NA | No question asked | NA | NA |
|  | **Food** | NA | No question asked | NA | NA |
|  | **Health** | New | Do you agree or disagree: Your household has access to a doctor/healthcare worker in a timely manner when you or someone in your household needs medical attention | Revised – Revised for clarity and to standardize how the respondent may define access and amount of access (your household has access vs. a majority of your household is able to see a doctor or healthcare worker…) | SO21 : Is a majority of your household able to see a doctor or healthcare worker when you or someone in your household needs medical attention? |
|  | **Preparation** | New | Do you agree or disagree: You and your household know what to do and where to go during a weather-related hazard. | Revised – Revised wording for clarity | SO25 Do you and your household know what to do and where to go before, during, and after a natural disaster? |
|  |  | New | Do you agree or disagree: Your household receives information about potential or predicted storms, floods, disasters, droughts etc., with enough time to plan and make decisions for the safety of your household and/or livelihoods. | Revised – Removed list to just ask about natural disasters to reduce confusion. | SO38 Your household receives information about potential or predicted natural disasters with enough time to plan and make decisions for the safety of your household. |
|  |  | New | Do you agree or disagree: I would know where to go to get information on how to protect myself and my household from environmental shocks and stressors such as typhoons, floods, droughts, or disease outbreaks. | Revised – Revised wording for clarity and removed unnecessary words. | SO39 I know where to find information on how to protect myself and my household from natural disasters. |
|  |  | New | Do you agree or disagree: Planning and preparing for natural disasters and weather-related hazards is a priority for my household. | Revised – Revised wording for clarity. | SO40 Planning and preparing for natural disasters is a priority for my household. |
|  |  | New |  | Added – We added this question to understand behavior around preparation. | SO23 Has your household prepared a disaster supply kit in case of emergencies which may include things such as cash, drinking water, food, flashlight, extra batteries, radio, whistle, local maps, etc.…? |
|  | **Rescue** | New | Do you agree or disagree: Your household is able to access safe shelter if you need to evacuate your home before or during weather related hazards. | Revised – Revised wording for clarity | SO22 If your household needs to evacuate because of a natural disaster, does your household has access to shelter that you feel safe going to such as [example]? |
|  | **Recovery** | New | Do you agree or disagree: You or someone in your household has knowledge or training on what to do before, during, and after a natural disaster or weather-related hazard. | Removed – We received feedback that the idea of “training” was not common. Additionally, we felt this question was similar to SO25. |  |

**Table S2: Age, gender, and economic satisfaction among resilience survey participants and non-participants**

|  | **Central Tendency** | | | **Test** | **P-value** |
| --- | --- | --- | --- | --- | --- |
|  | **Type** | **Participant**  **(n = 882)** | **Non-participant**  **(n = 514)** |  |  |
| Age (years) | Mean | 42.0 | 45.7 | 2-sample t-test | < 0.05 |
| Gender (female or male) | % Female | 82.4% | 67.4% | Chi-square test | < 0.05 |
| Economic Satisfaction  (scale 0 to 10 with 0: not satisfied – 10: most satisfied) | Median | 6 | 6 | 2-sample Wilcoxon Rank Sum test | < 0.05 |

######

###### **Table S3: Equivalence model fit parameters by scale**

Economic resilience scale model fit statistics are reported after dropping EC5, EC6, and EC10. Social resilience scale model fit statistics are reported after dropping SO22, SO23, SO25, SO27, SO33.

|  | **Model** | **CFI** | **TLI** | **SRMR** | **RMSEA Estimate** | **RMSEA Lower CI** | **RMSEA Higher CI** |
| --- | --- | --- | --- | --- | --- | --- | --- |
|  | **Criteria for ‘good’ fit** | **>0.95** | **>0.95** | **<0.08** | **<0.06** | | |
| **Economic Resilience Scale** | **Configural Model** | 0.987 | 0.991 | 0.103 | 0.312 | 0.285 | 0.341 |
|  | **Metric Model** | 0.988 | 0.993 | 0.085 | 0.276 | 0.216 | 0.251 |
|  | **Scalar Model** | 0.985 | 0.993 | 0.076 | 0.285 | 0.262 | 0.309 |
| **Social Resilience Scale** | **Configural Model** | 0.985 | 0.983 | 0.066 | 0.097 | 0.087 | 0.108 |
|  | **Metric Model** | 0.950 | 0.946 | 0.086 | 0.174 | 0.165 | 0.184 |
|  | **Scalar Model** | 0.984 | 0.983 | 0.074 | 0.097 | 0.087 | 0.106 |


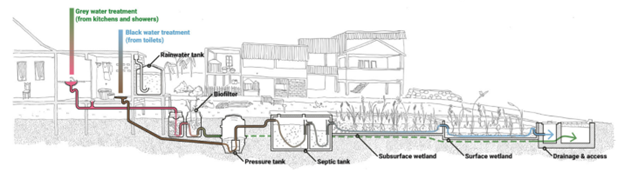


##### Fig S1: Schematic of proposed sanitation intervention adapted from Leder *et al*.^3^

##### Fig S2: Proportions of responses for ordinal economic resilience questions EC1-EC4

##### Fig S3: Proportions of responses for binary economic resilience questions EC5-EC10

##### Fig S4: Proportion of responses to ordinal environmental resilience questions EN11-EN20

##### Fig S5: Proportion of responses to social resilience questions SO21-SO25

##### Fig S6: Proportion of responses to social resilience question SO26-SO40


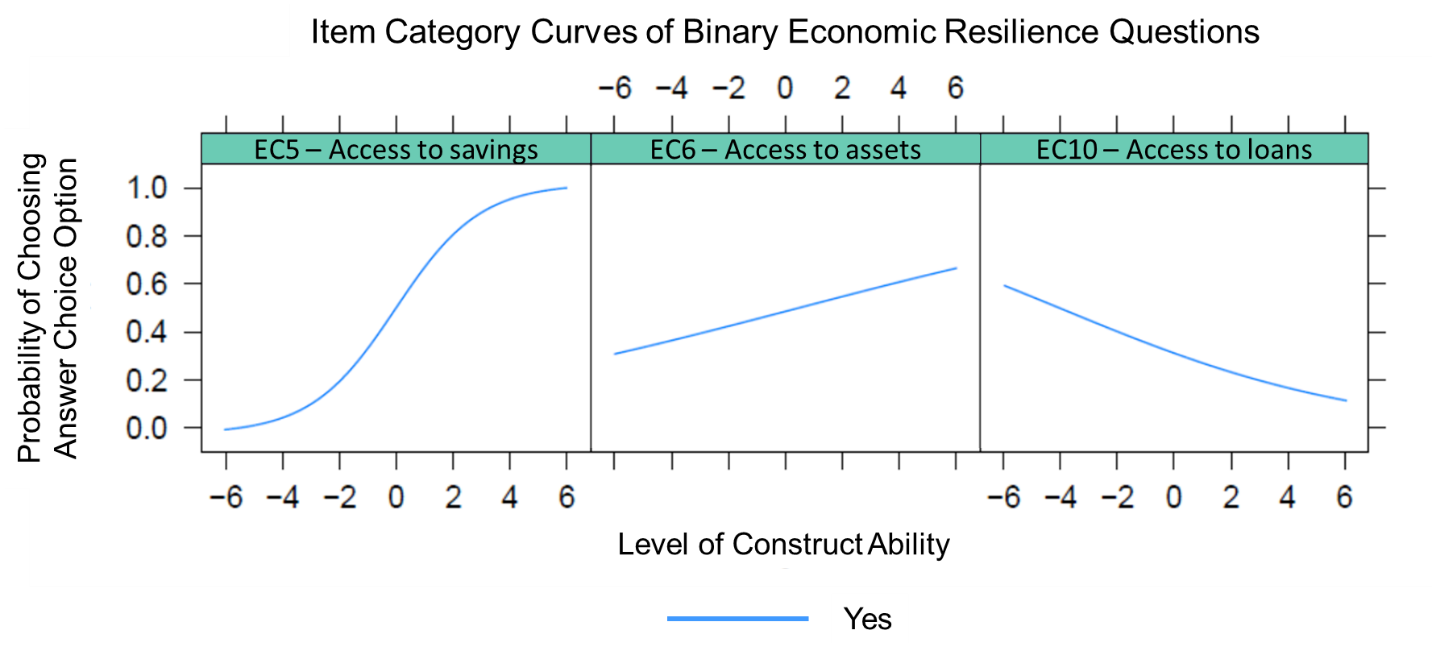


##### Fig S7: Binary economic resilience item category curves

Questions EC5-Access to savings, EC6-Access to assets, and EC10-Access to loans are binary questions with one ICC each, representing the probability of a respondent answering ‘yes’ for each level of the economic resilience construct. Question EC5 performed moderately well with an S-shaped curve indicating that those with higher levels of economic resilience (on the x-axis) are more likely (y-axis) to answer the questions to affirm economic resilience. Both EC6 and EC10 however, did not perform well. The ICCs for these two questions do not show a clear S-shaped curve with very low slopes, indicating the questions did not properly distinguish between those with low and high levels of economic resilience.


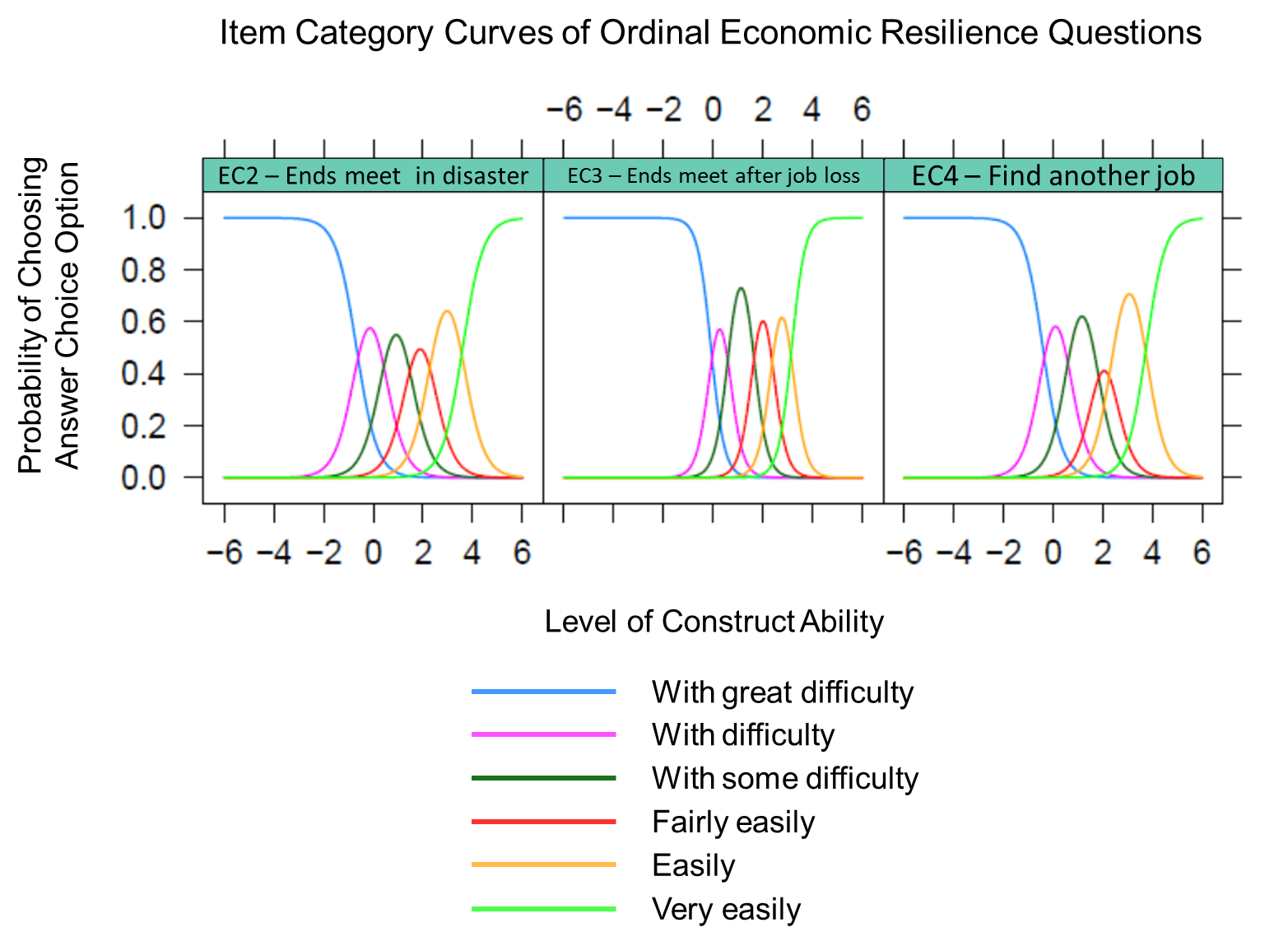


##### Fig S8: Ordinal economic resilience item category curves

Questions EC2-Ends meet in disaster, EC3-Ends meet after job loss, and EC4-Find another job contained six response options which are represented by the six ICCs. These three economic resilience questions performed well, each having distinct and clearly separated response curves for each response option.


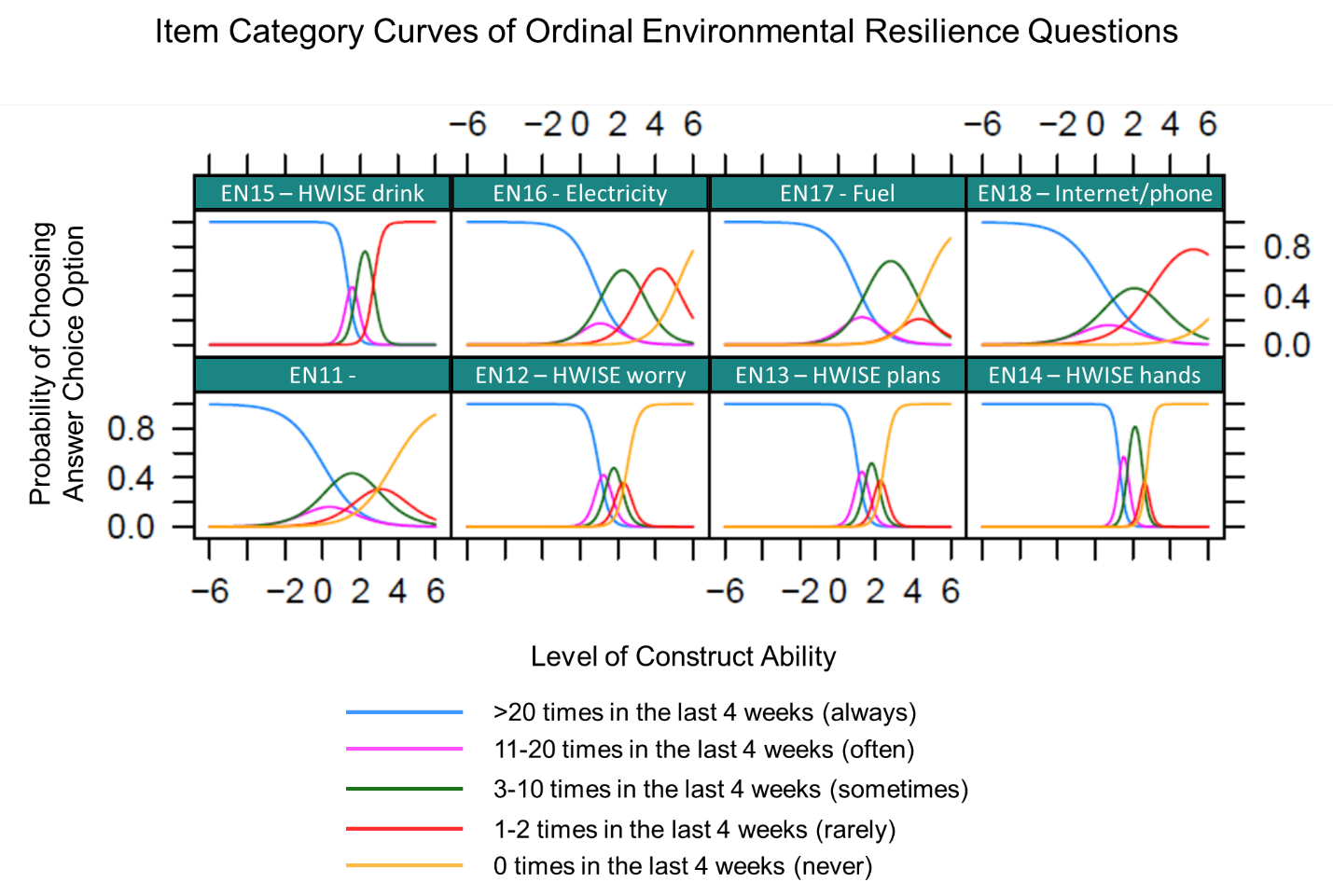


##### Fig S9 Environmental resilience item category curves

Questions EN11-EN18 contained five responses each represented by the five ICCs. Four of the eight questions, all within the infrastructure & energy factor (EN11; EN16-EN18) performed poorly, each having overlapping and indistinct response curves. This means the questions are not successfully differentializing among people with different levels of environmental resilience (indicated by theta). While the four items in the water sub-scale (which consisted of the four item HWISE short form scale^1^) performed well in the IRT analysis, we did not feel that this sub-scale alone would be appropriate for the whole environmental resilience scale. Therefore, we chose to drop the environmental scale entirely and recommended these set of questions be further refined. We have further discussed potential reasons for poor performance of the environmental scale and future directions to refine the scale in the discussion section.


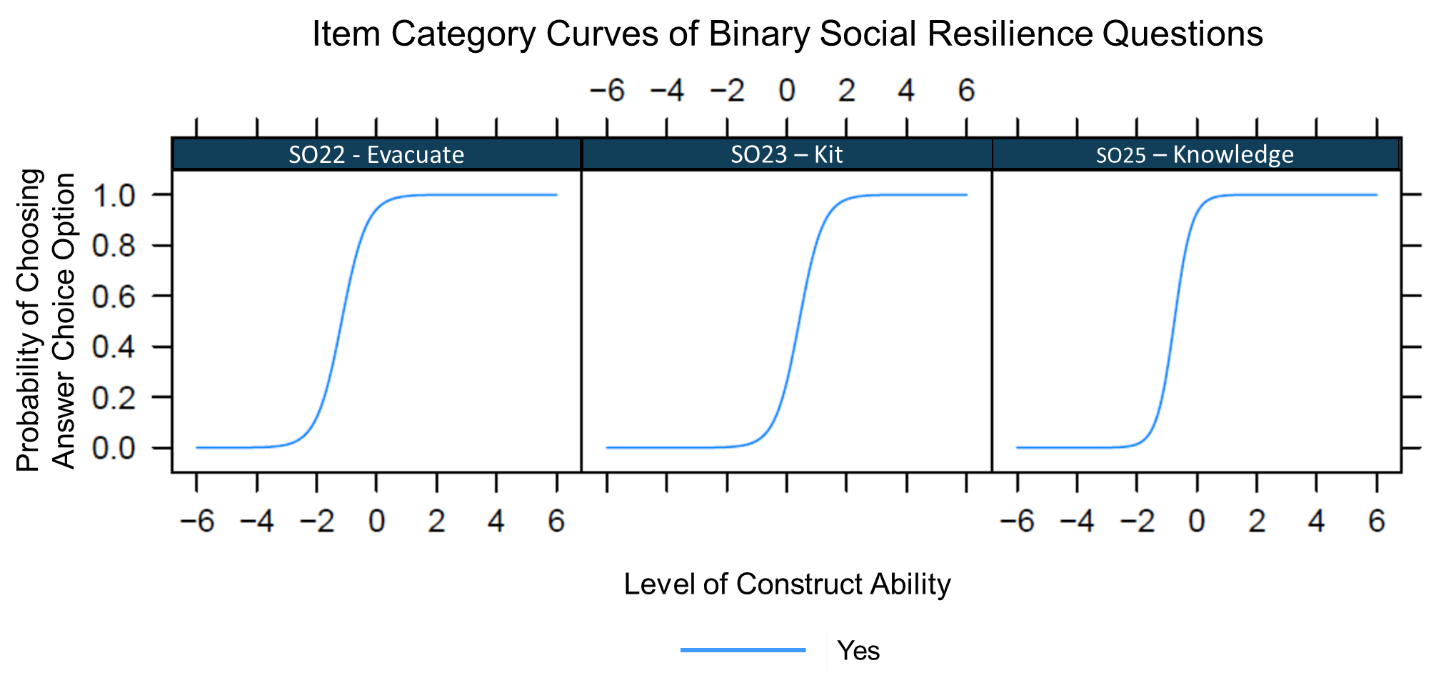


##### Fig S10 Ordinal social resilience item category curves

Items in the emergency response factor (SO22-Evacuate, SO23-Kit, SO25-Knowledge) performed well with an S-shaped curve indicating that those with higher levels of social resilience (on the x-axis) are more likely (y-axis) to answer the questions to affirm social resilience.


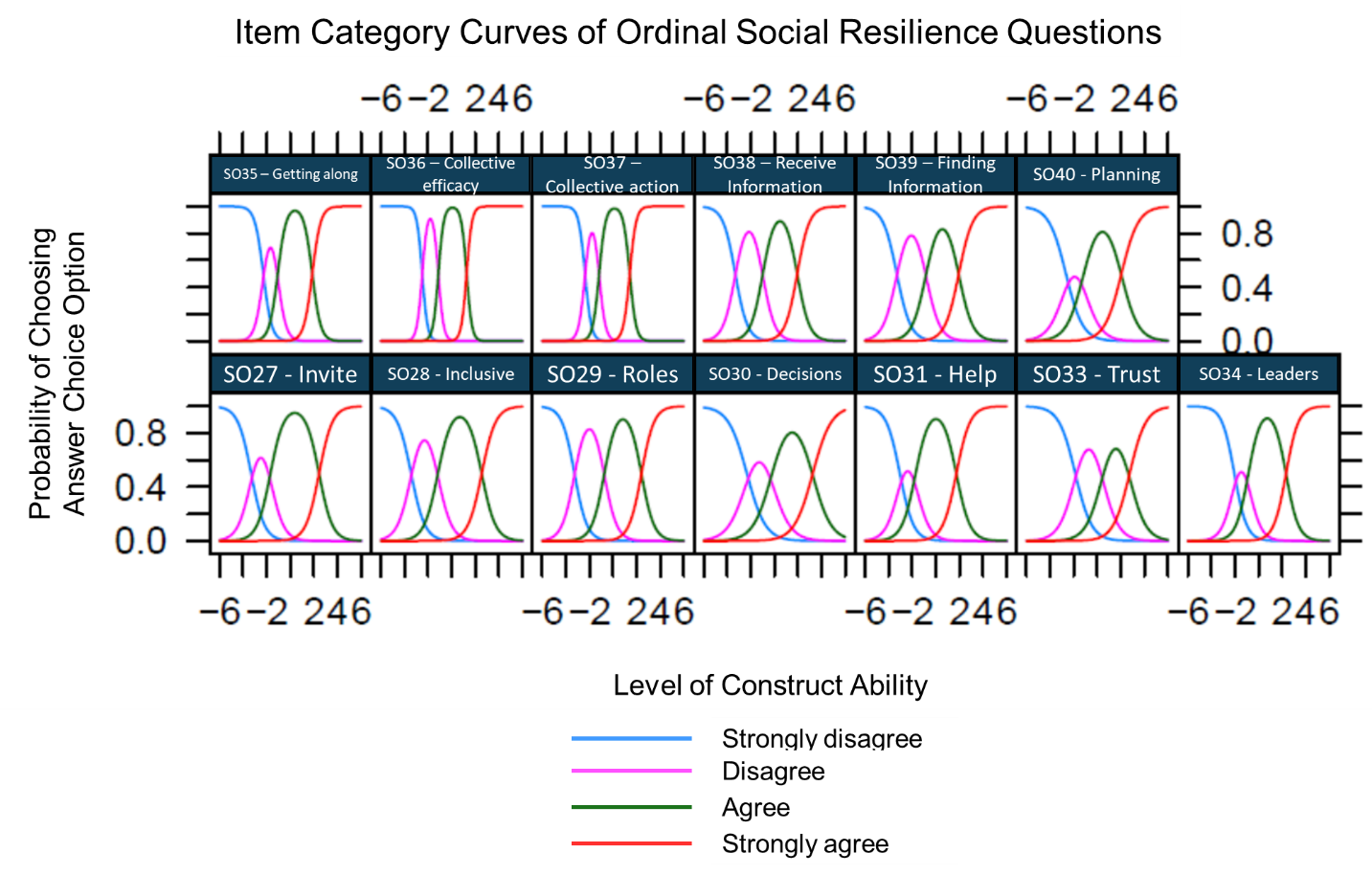


##### Fig S11 Ordinal social resilience item category curves

The remaining 13 questions contained four response options, each represented by the four ICCs per plot. These 13 social resilience questions performed well, each having distinct and clearly separated response curves for each response option. All 13 sets of curves indicated the response options successfully differentiated across varying degrees of social resilience.


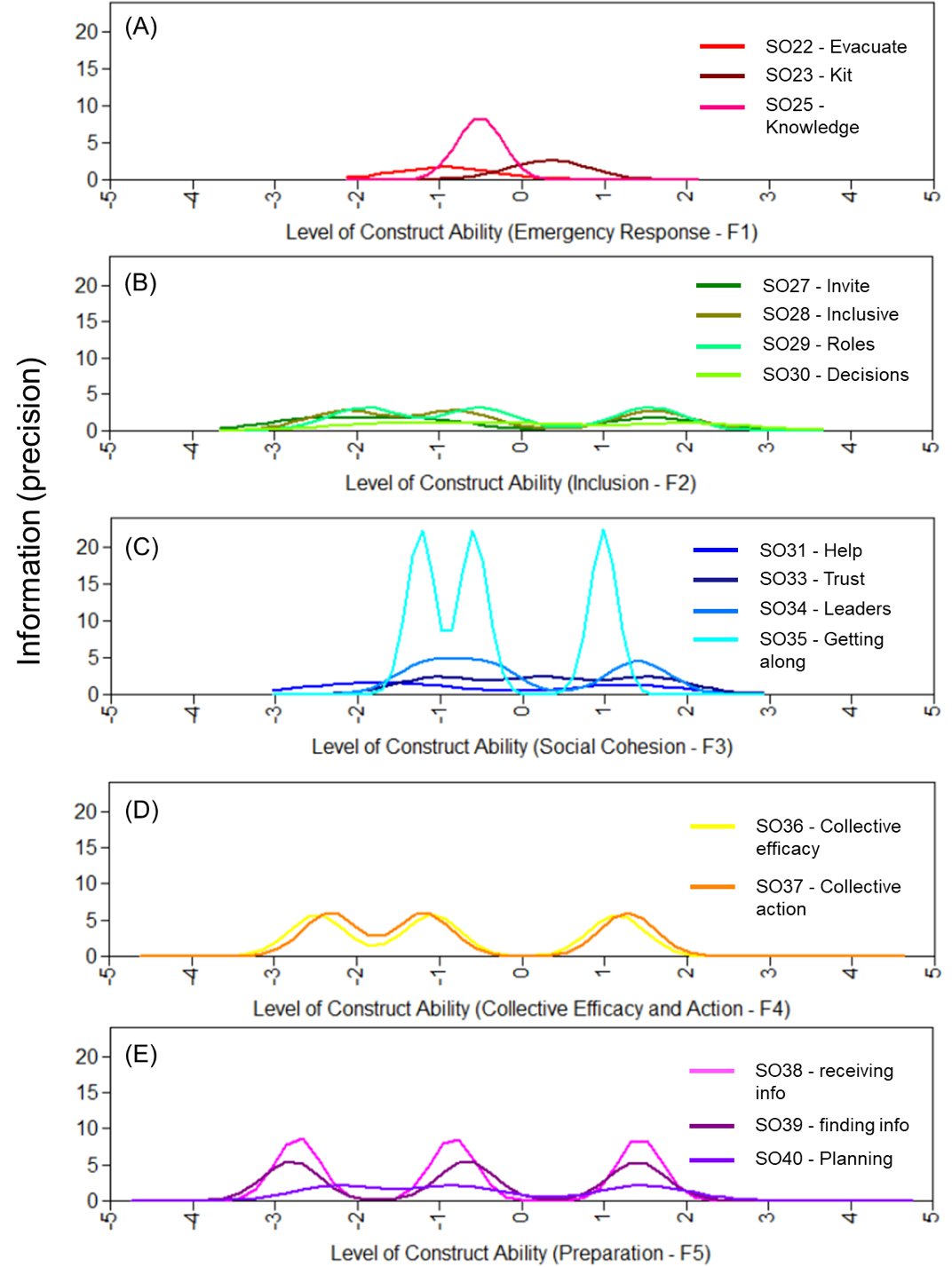
**Fig S12** Social resilience scale item information curve per item and by factor

The IICs provide insights into the relationship between the amount of information or measurement precision contributed (y-axis) at each level of the construct trait (x-axis). IICs for questions within the emergency response factor (A), inclusion factor (B), and collective efficacy and action factor (D) had relatively low vertical spread across the y-axis, meaning questions in among these factors had lower levels of precision. When comparing the horizontal spread on the x-axis, the inclusion factor (B) and collective efficacy and action factor (D) ICCs horizontal spread across the x-axis was larger than that of the emergency response (A) factor, indicating questions within the former two factors (B and D) provided information across a broader range of social resilience levels compared to those in the emergency response factor (A). Because the emergency response factor provided little information, or precision, over a relatively small range of the construct, we removed this factor and the corresponding three questions (SO22, SO23, and SO25) from the social resilience scale.


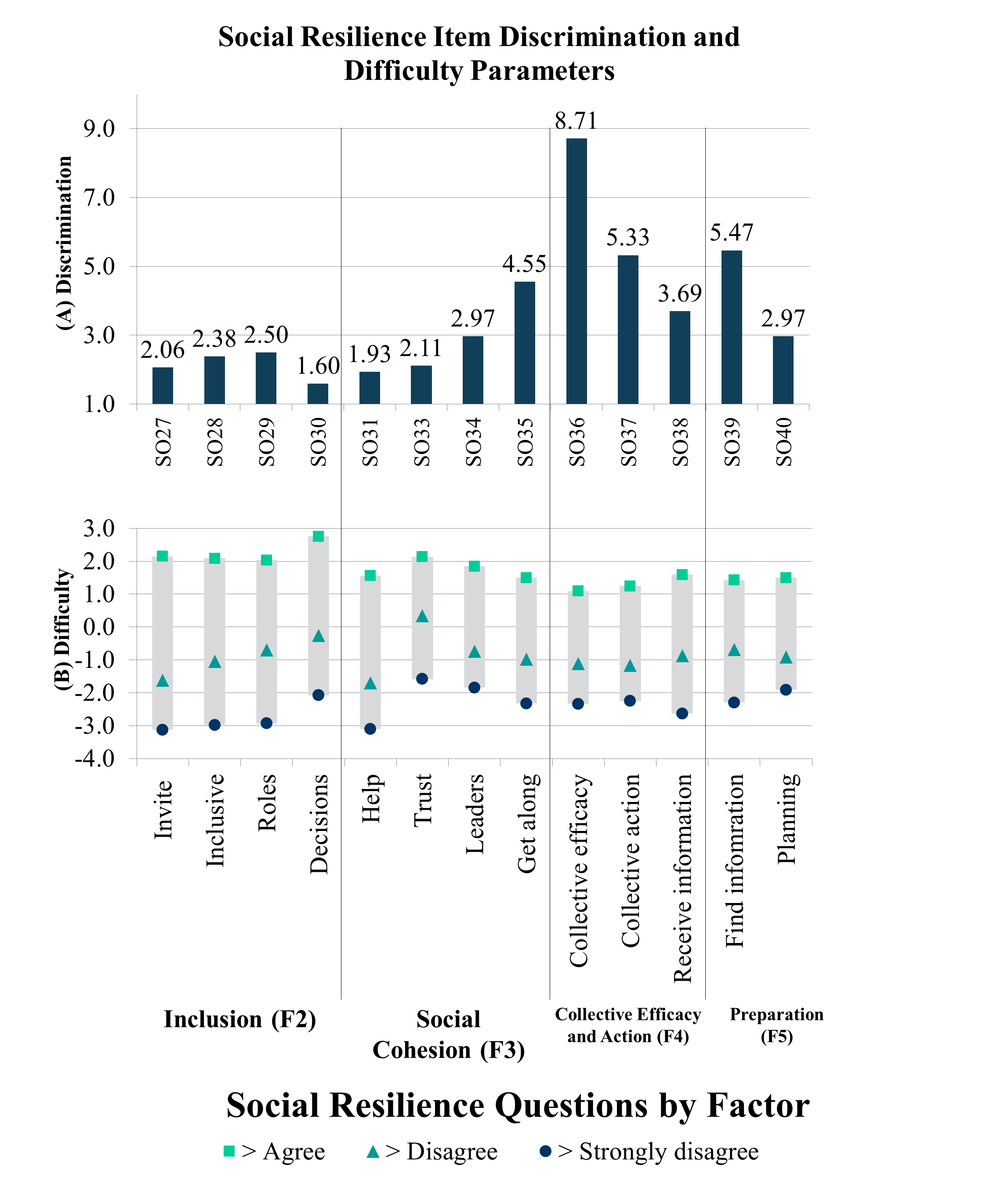


##### Fig S13: Social resilience item (A) discrimination and (B) difficulty parameters

The discrimination parameters indicate the ability of each question to differentiate respondents based on their level of social resilience with higher values per question indicating better discriminant ability.^2^ The discrimination parameters for questions within the full scale ranged from 1.60 to 8.71.

The difficulty ranges by question provides insights into the specific range of the trait that each question is capable of capturing. These assessments establish thresholds, or boundaries that divide the response options into distinct categories (represented by square, triangle, and circle markers on B). These thresholds translate the response options into different ranges of the trait.

In this study, questions within factors F2 to F5 were designed with four response options. The thresholds for these questions indicate the points where respondents are likely to transition from one response option to another based on their level of agreement with the question and, consequently, their position on the trait (displayed on the y-axis). For instance, consider question SO27 (invite), which covers levels of social resilience from approximately -3 to 2. If a respondent chooses to 'disagree' with this question, it generally implies that their position on the social resilience trait falls somewhere between -3 and -2. Conversely, answering 'agree' typically indicates a position on the social resilience trait ranging from -2 to 2.

We were unable to calculate the difficulty range for the three items pertaining to emergency response (SO22-evacuate, SO23-kit, and SO25-knowledge) due to their dichotomous response options. Considering their relatively poor performance illustrated in the IICs, we decided to drop these three items from the scale. These items may perform better after refinement and could be integrated into an environmental resilience domain instead.

The inclusion factor (F2) exhibited discrimination parameters ranging from 1.60 to 2.50, with the item on decisions (SO30) having the lowest discrimination level. However, SO30 accounted for the highest range of difficulty on the latent trait. We concluded that item SO27-invite, which displayed the second lowest discrimination level, was not essential to the measurement of the latent construct as its difficulty range on the latent trait was adequately covered by the remaining items in the scale.

The social cohesion factor (F3) exhibited discrimination parameters ranging from 1.93-4.55. The item on willingness to help a neighbor (SO31) had the lowest discrimination level, but also accounted for the lowest range of difficulty on the latent trait. Although items SO34 on leaders and SO35 on getting alone had overlapping difficulty ranges, their discrimination levels were relatively high, and SO34 was the only item that asked about thoughts on community leaders. Item SO33 on trust had the second lowest discrimination level. While it accounted for the highest difficulty range, after visually analyzing B, we felt that the range was only marginally higher than that of SO34 (leaders).

We did not consider reducing the collective efficacy and collective action factor (F4) since a factor must have at least two items. The items also demonstrated high discrimination levels ranging from 5.33-8.71, and a satisfactory spread of difficulty on the latent trait.

The preparation factor (F5) discrimination ranged from 2.97-5.47. Although SO40 on planning had the lowest discrimination level among items in this factor, it was the only item asking about prioritizing preparing for disturbances. Item SO38 on receiving information had the second lowest discrimination level but covered the largest range on the latent trait. Item SO39 on finding information had the highest discrimination level. We also felt that SO39 well represented household social resilience by asking if the respondents knew where to get information, rather than if their household received information from external sources. Because the items in F5 performed well, we opted to retain all three items.

**References**

1. Young, S. L. *et al.* Validity of a Four-Item Household Water Insecurity Experiences Scale for Assessing Water Issues Related to Health and Well-Being. *Am J Trop Med Hyg* 104, 391 (2021).

2. DeVellis, R. F. & Thorpe, C. T. *Scale Development: Theory and Applications Applied Social Research Methods*. (2021).

3. Leder, K. *et al.* Study design, rationale and methods of the Revitalising Informal Settlements and their Environments (RISE) study: a cluster randomised controlled trial to evaluate environmental and human health impacts of a water-sensitive intervention in informal settle. *BMJ Open* 11, e042850 (2021).
