# Supplementary material for: Resilience measurement for environmental shocks and stressors: scale development and psychometric assessment for coastal urban informal settlements in Fiji and Indonesia
Source: BMC Glob Public Health. 2025 Jul 9;3:61. doi: 10.1186/s44263-025-00177-3 (PMC12243337; doi:10.1186/s44263-025-00177-3)
Supplement: Supplementary file 2 — Additional file 2. Cognitive interview guide. [file 44263_2025_177_MOESM2_ESM.docx]

**RISE | Resilience Measurement Study**

Cognitive Interview Guide

**Date**: ________________________, ______________________ ______, ____________

Day of Week Month Day Year

**Interviewer Name**: _______________________________________

**Notetaker Name**: ________________________________________

**Responder Name**: _______________________________________

**Start Time**: _____ : _____ (AM / PM)

Hour Minute

**End Time**: _____ : _____ (AM / PM)

Hour Minute

Purpose of Cognitive Interview

Hello, my name is Hemali Oza. I am doing research at Emory University in Atlanta, Georgia, in the United States. I am working on a project to measure household and community resilience to environmental hazards such as storms, floods, and droughts. I have invited you for an interview to inform how to better write my survey questions that will be added to the September data collection activity. In total, I will be interviewing about 6 members of the Indonesia team and 10 members of the Fiji team.

Methods and procedures

If you agree to participate, I would like to interview you. This interview will start with me reading you a series of statements. After I read each statement, I will ask you what you think about them. We would like to know your views about the statements in our survey, especially what they mean to you, whether they make sense, and how difficult or easy they are to answer. Some of the statements and follow-up questions might sound a little unusual. For example, I might ask you what a certain word means to you. There are no right or wrong answers. I am more interested in how you came to your answer than the answer itself.

Length of Interview

The interview will take about 60 to 90 minutes.

Future use of your interview answers

I will use your answers to revise the survey questions to make them easier to understand and answer. I will then interview another staff member tomorrow with the revised questions. I will continue this process until I feel that the questions are well understood, make sense to the responders, and are easy to answer.

Right not to participate

Taking part in this interview is voluntary. You can choose not to be interviewed at all, and if you do agree to be interviewed, you can stop the interview at any time if you wish, or skip any questions that you do not want to answer. There are no right or wrong answers. We just want to hear more about your own ideas and beliefs.

Do you have any questions? Is there anything I said that you did not understand? Would you like me to repeat anything?

Do you agree to take part in the interview?

[ ] Yes

[ ] No → Thank you for your time! [End interview]

Cognitive Interview - Respondent Training

Now, if you’re ready, I’d like to start by reading you some survey prompts and asking you some questions about them. As I said earlier, there are no right or wrong answers. After I read each prompt, I will ask you to “think aloud” as you respond so that I can learn what comes to your mind as you give your response to the prompt. After I read certain prompts, I will ask you to restate the prompt in your own words or to tell me about the meaning of some words used in the prompt. Finally, when we are done, I will ask you your general opinion of the survey prompts.

Do you have any questions? [pause and answer].

Example 1:

Before we move on to the actual survey prompts, I would like to start with two practice prompts, each that has a different set of response options. I will start by reading you a prompt aloud along with answer choices. As you think of your response, I would like you to talk “out loud” and walk me through what you are thinking. Again, I am most interested in how you arrive at your answer.

I will read a statement to you, and ask you to indicate your level of agreement with the statement: whether you completely disagree, partly disagree, neither disagree nor agree (i.e., feel neutral), partly agree, or completely agree with the statement.

“A motorcycle has four wheels.” Do you…

[ ] Strongly agree

[ ] Agree

[ ] Neither agree or disagree

[ ] Disagree

[ ] Strongly disagree

PROBE: What comes to mind when I say “motorcycle”?

____________________________________________________________________________

PROBE: Can you tell me how you got to your answer?

__________________________________________________________________________________________________________________________________________________________________________

Thank you for completing that exercise with me. Now that you understand what I would like you to do as we go through each survey prompt, let’s begin.

Interview Probe Cheat Sheet

GENERAL PROBES (USE AS NECESSARY):

1. Please tell me more about what you are thinking.

2. How did you choose your response, or how did you arrive at that response?

3. Could you tell me more about that?

4. I noticed you [smiled/frowned/paused] while answering. Could you tell me what you were thinking?

5. How difficult was it to indicate your response to this statement?

6. If the participant doesn’t know or doesn’t remember, the Interviewer says: “Can you explain to me why you don’t know or don’t remember?”

7. If the participant refuses to indicate a response, the Interviewer says: “You do not need to respond. If you are comfortable, can you share with me why you do not want to indicate your response for this prompt?”

8. Be aware of contradictions or inconsistencies in answers and follow-up if answers don’t match up.

| Section 1.1 Economic Resilience: Livelihoods | | |
| --- | --- | --- |
| Now I am going to ask you questions about your household’s income. Some questions may ask about family members specifically. Additionally, some questions may talk about ‘environmental shocks and stressors.’ This refers to weather related hazards such as typhoons, storms, floods, or droughts, or disease outbreaks like the COVID-19 pandemic. | | |
| Item # | Statement: Income Level | Answer Choices |
| EC.1 | Thinking of your household's total monthly income or weekly income, how difficult is it for your household to make ends meet, that is pay your usual expenses? | [ ] With great difficulty [ ] Fairly easily  [ ] With difficulty [ ] Easily  [ ] With some difficulty [ ] Very easily |
| Probe | What does this mean to you? How would you put in your own words? | If participant does not understand, how was it described by the interviewer?  Was this explanation successful? [ YES / NO ] |
| Type of question failure | [ ] Issues with sequencing, length or sensitivity [ ] Problematic response options  [ ] Inappropriate vocabulary and long sentences [ ] Temporal and spatial confusion  [ ] Accessing different cognitive domains [ ] Failure to resonate with the respondent’s world  view and reality | |
| Notes for Revisions |  | |
| Item # | Statement: Sustainable Income | Answer Choices |
| EC.2 | How difficult is it for your househod to make ends meet after an environmental shocks and stressors such as a typhoon, flood, or disease outbreak? | [ ] With great difficulty [ ] Fairly easily  [ ] With difficulty [ ] Easily  [ ] With some difficulty [ ] Very easily |
| Probe | What does this mean to you? How would you put in your own words?  How did you come to your answer?  What comes to mind when I say environmental hazards? | If the participant does not understand, how was it described by the interviewer?  Was this explanation successful? [ YES / NO ] |
| Type of question failure | [ ] Issues with sequencing, length or sensitivity [ ] Problematic response options  [ ] Inappropriate vocabulary and long sentences [ ] Temporal and spatial confusion  [ ] Accessing different cognitive domains [ ] Failure to resonate with the respondent’s world  view and reality | |
| Notes for Revisions |  | |

| Item # | Statement: Sustainable Income | Answer Choices |
| --- | --- | --- |
| EC.3 | If someone in your household loses their job, how difficult is it to make ends meet? | [ ] With great difficulty [ ] Fairly easily  [ ] With difficulty [ ] Easily  [ ] With some difficulty [ ] Very easily |
| Probe | What does this mean to you? How would you put it in your own words?  How did you come to your answer? | If the participant does not understand, how was it described by the interviewer?  Was this explanation successful? [ YES / NO ] |
| Type of question failure | [ ] Issues with sequencing, length or sensitivity [ ] Problematic response options  [ ] Inappropriate vocabulary and long sentences [ ] Temporal and spatial confusion  [ ] Accessing different cognitive domains [ ] Failure to resonate with the respondent’s world  view and reality | |
| Notes for Revisions |  | |
| Item # | Statement: Diversification of Income | Answer Choices |
| EC.4 | How difficult is it for the main income earner to find another job if they lost their current job? | [ ] With great difficulty [ ] Fairly easily  [ ] With difficulty [ ] Easily  [ ] With some difficulty [ ] Very easily |
| Probe | What does this mean to you? How would you put it in your own words? | If the participant does not understand, how was it described by the interviewer?  Was this explanation successful? [ YES / NO ] |
| Type of question failure | [ ] Issues with sequencing, length or sensitivity [ ] Problematic response options  [ ] Inappropriate vocabulary and long sentences [ ] Temporal and spatial confusion  [ ] Accessing different cognitive domains [ ] Failure to resonate with the respondent’s world  view and reality | |
| Notes for Revisions |  | |

| Section 1.2 Economic Resilience: Financial Resources | | |
| --- | --- | --- |
| Now I am going to ask you questions about other financial resources that your household may have. Please indicate how strongly you agree or disagree with the statements. | | |
| Item # | Statement: Savings | Answer Choices |
| EC.5 | During or after environmental shocks and stressors such as typhoons, floods, droughts, or disease outbreaks, your household hasaccess to savings that could be used to help support you and your household. | [ ] Strongly agree [ ] Disagree  [ ] Agree [ ] Strongly disagree  [ ] Neither agree or disagree |
| Probe | What does this mean to you? How would you put it in your own words?  What does ‘back to normal’ mean to you?  *may need additional probing* | If the participant does not understand, how was it described by the interviewer?  Was this explanation successful? [ YES / NO ] |
| Type of question failure | [ ] Issues with sequencing, length or sensitivity [ ] Problematic response options  [ ] Inappropriate vocabulary and long sentences [ ] Temporal and spatial confusion  [ ] Accessing different cognitive domains [ ] Failure to resonate with the respondent’s world  view and reality | |
| Notes for Revisions |  | |
| Item # | Statement: Assets | Answer Choices |
| EC.6 | Your household has assets that could be sold for additional income if needed. This may include household assets or other assets such a livestock. | [ ] Strongly agree [ ] Disagree  [ ] Agree [ ] Strongly disagree  [ ] Neither agree or disagree |
| Probe | What does this mean to you? How would you put it in your own words?  What do you think of when I say assets? | If the participant does not understand, how was it described by the interviewer?  Was this explanation successful? [ YES / NO ] |
| Type of question failure | [ ] Issues with sequencing, length or sensitivity [ ] Problematic response options  [ ] Inappropriate vocabulary and long sentences [ ] Temporal and spatial confusion  [ ] Accessing different cognitive domains [ ] Failure to resonate with the respondent’s world  view and reality | |
| Notes for Revisions |  | |

| Item # | Statement: Credit | Answer Choices |
| --- | --- | --- |
| EC.7 | If your household is need of additional income, you or someone in your household has access to money transfers from outside your community or abroad. | [ ] Strongly agree [ ] Disagree  [ ] Agree [ ] Strongly disagree  [ ] Neither agree or disagree |
| Probe | What does this mean to you? How would you put it in your own words?  What comes to mind when I say money transfer? | If the participant does not understand, how was it described by the interviewer?  Was this explanation successful? [ YES / NO ] |
| Type of question failure | [ ] Issues with sequencing, length or sensitivity [ ] Problematic response options  [ ] Inappropriate vocabulary and long sentences [ ] Temporal and spatial confusion  [ ] Accessing different cognitive domains [ ] Failure to resonate with the respondent’s world  view and reality | |
| Notes for Revisions |  | |
| Item # | Statement: Aid | Answer Choices |
| EC.8 | After environmental shocks or stressors, you or someone in your household (including children) receive aid or assistance such as food, cash, or other support. This could include assistance from local, regional, or national government, non-profits, religious organizations, non-governmental organizations, or other service groups. | [ ] Strongly agree [ ] Disagree  [ ] Agree [ ] Strongly disagree  [ ] Neither agree or disagree |
| Probe | What does this mean to you? How would you put it in your own words?  What types of ‘other support’ were you thinking of?  What did you think of when I said ‘usually’? Did that make the question more difficult to answer? | If the participant does not understand, how was it described by the interviewer?  Was this explanation successful? [ YES / NO ] |
| Type of question failure | [ ] Issues with sequencing, length or sensitivity [ ] Problematic response options  [ ] Inappropriate vocabulary and long sentences [ ] Temporal and spatial confusion  [ ] Accessing different cognitive domains [ ] Failure to resonate with the respondent’s world  view and reality | |
| Notes for Revisions |  | |

| Section 2.1 Environmental Resilience: Built Environment | | |
| --- | --- | --- |
| Now I will ask questions about your surroundings like your housing and roads. I’ll also ask questions about utilities like water and electricity. As mentioned before, some questions may talk about ‘environmental shocks and stressors.’ This refers to weather related hazards such as typhoons, storms, floods, or droughts, or disease outbreaks like the COVID-19 pandemic. I will ask how many times in the last 4 weeks something has happened or you have thought of something. Answer to the best of your ability. | | |
| Item # | Statement: Housing Quality | Answer Choices |
| EN.1 | In the last 4 weeks, how many times did you worry about the quality or structural integrity of your house? | [ ] Never (0 times)  [ ] Rarely (1-2 time)  [ ] Sometimes (3-10 times)  [ ] Often (11-20 times)  [ ] Always (more than 20 times) |
| Probe | What does this mean to you? How would you put it in your own words?  What do you think of when I say structural integrity? | If the participant does not understand, how was it described by the interviewer?  Was this explanation successful? [ YES / NO ] |
| Type of question failure | [ ] Issues with sequencing, length or sensitivity [ ] Problematic response options  [ ] Inappropriate vocabulary and long sentences [ ] Temporal and spatial confusion  [ ] Accessing different cognitive domains [ ] Failure to resonate with the respondent’s world  view and reality | |
| Notes for Revisions |  | |
| Item # | Statement: Water Security | Answer Choices |
| EN.2 | HWISE_WORRY: In the last 4 weeks, how frequently did you or anyone in your household (including children) worry you would not have enough water for all of your household needs? | [ ] Never (0 times)  [ ] Rarely (1-2 time)  [ ] Sometimes (3-10 times)  [ ] Often (11-20 times)  [ ] Always (more than 20 times) |
| Probe | What does this mean to you? How would you put it in your own words?  Which household needs were you considering when you were coming up with your answer? | If the participant does not understand, how was it described by the interviewer?  Was this explanation successful? [ YES / NO ] |
| Type of question failure | [ ] Issues with sequencing, length or sensitivity [ ] Problematic response options  [ ] Inappropriate vocabulary and long sentences [ ] Temporal and spatial confusion  [ ] Accessing different cognitive domains [ ] Failure to resonate with the respondent’s world  view and reality | |
| Notes for Revisions |  | |
| Item # | Statement: Water Security | Answer Choices |
| EN.3 | HWISE_PLANS: In the last 4 weeks, how frequently have you or anyone in your household (including children) had to change activities or timing of activities due to problems with your water situation? | [ ] Never (0 times)  [ ] Rarely (1-2 time)  [ ] Sometimes (3-10 times)  [ ] Often (11-20 times)  [ ] Always (more than 20 times) |
| Probe | What does this mean to you? How would you put it in your own words?  If >’never’, Can you give me an example of a scenario you were thinking of?  Did you have any difficulty remembering how often this happened in the last 4 weeks? Why/why not? What did you do to remind yourself/help yourself remember (e.g., temporal references such as holidays, thinking of last week and multiplying by 4)? | If the participant does not understand, how was it described by the interviewer?  Was this explanation successful? [ YES / NO ] |
| Type of question failure | [ ] Issues with sequencing, length or sensitivity [ ] Problematic response options  [ ] Inappropriate vocabulary and long sentences [ ] Temporal and spatial confusion  [ ] Accessing different cognitive domains [ ] Failure to resonate with the respondent’s world  view and reality | |
| Notes for Revisions |  | |

| Item # | Statement: Water Security | Answer Choices |
| --- | --- | --- |
| EN.4 | HWISE_HANDS: In the last 4 weeks, how frequently have you or anyone in your household (*including children) were not able to wash hands after dirty activities (e.g., defecating or changing diapers, cleaning animal dung) because of problems with water? | [ ] Never (0 times)  [ ] Rarely (1-2 time)  [ ] Sometimes (3-10 times)  [ ] Often (11-20 times)  [ ] Always (more than 20 times) |
| Probe | What does this mean to you? How would you put it in your own words? | If the participant does not understand, how was it described by the interviewer?  Was this explanation successful? [ YES / NO ] |
| Type of question failure | [ ] Issues with sequencing, length or sensitivity [ ] Problematic response options  [ ] Inappropriate vocabulary and long sentences [ ] Temporal and spatial confusion  [ ] Accessing different cognitive domains [ ] Failure to resonate with the respondent’s world  view and reality | |
| Notes for Revisions |  | |
| Item # | Statement: Water Security | Answer Choices |
| EN.5 | HWISE_DRINK: in the last 4 weeks, how frequently has there not been as much water to drink as you would like for you or anyone in your household (including children)? | [ ] Never (0 times)  [ ] Rarely (1-2 time)  [ ] Sometimes (3-10 times)  [ ] Often (11-20 times)  [ ] Always (more than 20 times) |
| Probe | What does this mean to you? How would you put it in your own words? | If the participant does not understand, how was it described by the interviewer?  Was this explanation successful? [ YES / NO ] |
| Type of question failure | [ ] Issues with sequencing, length or sensitivity [ ] Problematic response options  [ ] Inappropriate vocabulary and long sentences [ ] Temporal and spatial confusion  [ ] Accessing different cognitive domains [ ] Failure to resonate with the respondent’s world  view and reality | |
| Notes for Revisions |  | |

| Item # | Statement: Electricity | Answer Choices |
| --- | --- | --- |
| EN.6 | In the last 4 weeks, how frequently did you or anyone in your household worry that your main source of electricity, internet, phone, or gas may stop working? | [ ] Never (0 times)  [ ] Rarely (1-2 time)  [ ] Sometimes (3-10 times)  [ ] Often (11-20 times)  [ ] Always (more than 20 times) |
| Probe | What does this mean to you? How would you put it in your own words?  What do you think of when I say interruption? | If the participant does not understand, how was it described by the interviewer?  Was this explanation successful? [ YES / NO ] |
| Type of question failure | [ ] Issues with sequencing, length or sensitivity [ ] Problematic response options  [ ] Inappropriate vocabulary and long sentences [ ] Temporal and spatial confusion  [ ] Accessing different cognitive domains [ ] Failure to resonate with the respondent’s world  view and reality | |
| Notes for Revisions |  | |
| Item # | Statement: Transportation | Answer Choices |
| EN.7 | In the last 4 weeks, how many times were you unable to access transportation to get to somewhere you needed to be? | [ ] Never (0 times)  [ ] Rarely (1-2 time)  [ ] Sometimes (3-10 times)  [ ] Often (11-20 times)  [ ] Always (more than 20 times) |
| Probe | What does this mean to you? How would you put it in your own words?  How did you differentiate between times you needed to be somewhere and times you wanted to be somewhere, if at all? | If the participant does not understand, how was it described by the interviewer?  Was this explanation successful? [ YES / NO ] |
| Type of question failure | [ ] Issues with sequencing, length or sensitivity [ ] Problematic response options  [ ] Inappropriate vocabulary and long sentences [ ] Temporal and spatial confusion  [ ] Accessing different cognitive domains [ ] Failure to resonate with the respondent’s world  view and reality | |
| Notes for Revisions |  | |

| Item # | Statement: Roads | Answer Choices |
| --- | --- | --- |
| EN.8 | In the last 4 weeks, how many times were you concerned that you would not be able to use the roads and/or accessways to get to where you needed to be? This may have been becuase of floods or high water levels, obstacles in your path, or damage to the road that made it unusable. | [ ] Never (0 times)  [ ] Rarely (1-2 time)  [ ] Sometimes (3-10 times)  [ ] Often (11-20 times)  [ ] Always (more than 20 times) |
| Probe | What does this mean to you? How would you put it in your own words?  What do roads and accesssways mean to you? | If the participant does not understand, how was it described by the interviewer?  Was this explanation successful? [ YES / NO ] |
| Type of question failure | [ ] Issues with sequencing, length or sensitivity [ ] Problematic response options  [ ] Inappropriate vocabulary and long sentences [ ] Temporal and spatial confusion  [ ] Accessing different cognitive domains [ ] Failure to resonate with the respondent’s world  view and reality | |
| Notes for Revisions |  | |

| Section 3.1 Social Resilience: Services | | |
| --- | --- | --- |
| Now I am going to ask you about access to services like healthcare, political environments, knowledge on weather related hazards, and general community connections. As mentioned before, some questions may talk about ‘environmental shocks and stressors.’ This refers to weather related hazards such as typhoons, storms, floods, or droughts, or disease outbreaks like the COVID-19 pandemic. Listen to the statement and tell me how strongly you agree or disagree. | | |
| Item # | Statement: Healthcare | Answer Choices |
| SO.1 | Your household has access to a doctor/healthcare worker in a timely manner when you or someone in your household needs medical attention | [ ] Strongly agree [ ] Disagree  [ ] Agree [ ] Strongly disagree  [ ] Neither agree or disagree |
| Probe | What does this mean to you? How would you put it in your own words?  When you seek medical attention, what do you usually do?  How did you decide what was ‘easy’?  Does this change based on the shock or stressor? | If the participant does not understand, how was it described by the interviewer?  Was this explanation successful? [ YES / NO ] |
| Type of question failure | [ ] Issues with sequencing, length or sensitivity [ ] Problematic response options  [ ] Inappropriate vocabulary and long sentences [ ] Temporal and spatial confusion  [ ] Accessing different cognitive domains [ ] Failure to resonate with the respondent’s world  view and reality | |
| Notes for Revisions |  | |
| Item # | Statement: Emergency Response | Answer Choices |
| SO.2 | Your household is able to access safe shelter if you need to evacuate your home beofre or during weather related hazards. | [ ] Strongly agree [ ] Disagree  [ ] Agree [ ] Strongly disagree  [ ] Neither agree or disagree |
| Probe | What does this mean to you? How would you put it in your own words?  Can you tell me what safe shelter and access means to you? | If the participant does not understand, how was it described by the interviewer?  Was this explanation successful? [ YES / NO ] |
| Type of question failure | [ ] Issues with sequencing, length or sensitivity [ ] Problematic response options  [ ] Inappropriate vocabulary and long sentences [ ] Temporal and spatial confusion  [ ] Accessing different cognitive domains [ ] Failure to resonate with the respondent’s world  view and reality | |
| Notes for Revisions |  | |
| Item # | Statement: Emergency Response | Answer Choices |
| SO.3 | You and your household are able to access authorities, emergency rescue, or medical aid during weather related hazards. | [ ] Strongly agree [ ] Disagree  [ ] Agree [ ] Strongly disagree  [ ] Neither agree or disagree |
| Probe | What does this mean to you? How would you put it in your own words?  What lead you to your answer?  What type of threat or hazard were you thinking about when answering this question?  Does this change based on the type of threat or hazard? | If the participant does not understand, how was it described by the interviewer?  Was this explanation successful? [ YES / NO ] |
| Type of question failure | [ ] Issues with sequencing, length or sensitivity [ ] Problematic response options  [ ] Inappropriate vocabulary and long sentences [ ] Temporal and spatial confusion  [ ] Accessing different cognitive domains [ ] Failure to resonate with the respondent’s world  view and reality | |
| Notes for Revisions |  | |
| Item # | Statement: Emergency Response | Answer Choices |
| SO.4 | You and your household know what to do and where to go during a weather realated hazards. | [ ] Strongly agree [ ] Disagree  [ ] Agree [ ] Strongly disagree  [ ] Neither agree or disagree |
| Probe | What does this mean to you? How would you put it in your own words?  What lead you to your answer? | If the participant does not understand, how was it described by the interviewer?  Was this explanation successful? [ YES / NO ] |
| Type of question failure | [ ] Issues with sequencing, length or sensitivity [ ] Problematic response options  [ ] Inappropriate vocabulary and long sentences [ ] Temporal and spatial confusion  [ ] Accessing different cognitive domains [ ] Failure to resonate with the respondent’s world  view and reality | |
| Notes for Revisions |  | |

| Section 3.2 Social Resilience: Community and Institutional Environments | | |
| --- | --- | --- |
| Item # | Statement: Political Environment | Answer Choices |
| SO.5 | When most of the leaders of this settlement make decisions/policies, they are accepted and good for most households in your settlement. | [ ] Strongly agree [ ] Disagree  [ ] Agree [ ] Strongly disagree  [ ] Neither agree or disagree |
| Probe | What does this mean to you? How would you put it in your own words?  What do you think of when I say leaders?  When I say leaders, at what level are you thinking? | If the participant does not understand, how was it described by the interviewer?  Was this explanation successful? [ YES / NO ] |
| Type of question failure | [ ] Issues with sequencing, length or sensitivity [ ] Problematic response options  [ ] Inappropriate vocabulary and long sentences [ ] Temporal and spatial confusion  [ ] Accessing different cognitive domains [ ] Failure to resonate with the respondent’s world  view and reality | |
| Notes for Revisions |  | |

| Item # | Statement: Political Environment | Answer Choices |
| --- | --- | --- |
| SO.6 | You and your household have access to the authorities, local government, community leaders who are in charge of the decisions that impact your day to day lives. | [ ] Strongly agree [ ] Disagree  [ ] Agree [ ] Strongly disagree  [ ] Neither agree or disagree |
| Probe | What does this mean to you? How would you put it in your own words?  What does it mean to have access to someone in charge? | If the participant does not understand, how was it described by the interviewer?  Was this explanation successful? [ YES / NO ] |
| Type of question failure | [ ] Issues with sequencing, length or sensitivity [ ] Problematic response options  [ ] Inappropriate vocabulary and long sentences [ ] Temporal and spatial confusion  [ ] Accessing different cognitive domains [ ] Failure to resonate with the respondent’s world  view and reality | |
| Notes for Revisions |  | |

| Item # | Statement: Inclusion | Answer Choices |
| --- | --- | --- |
| SO.7 | When there is a community events, gatherings, trainings, or meetings, someone from my household is usually invited to attend. | [ ] Strongly agree [ ] Disagree  [ ] Agree [ ] Strongly disagree  [ ] Neither agree or disagree |
| Probe | What does this mean to you? How would you put it in your own words?  What do you think of when I say community events, gatherings, trainings, or meetings? | If the participant does not understand, how was it described by the interviewer?  Was this explanation successful? [ YES / NO ] |
| Type of question failure | [ ] Issues with sequencing, length or sensitivity [ ] Problematic response options  [ ] Inappropriate vocabulary and long sentences [ ] Temporal and spatial confusion  [ ] Accessing different cognitive domains [ ] Failure to resonate with the respondent’s world  view and reality | |
| Notes for Revisions |  | |
| Item # | Statement: Inclusion | Answer Choices |
| SO.8 | Some community members have pre-defined responsibilities or jobs that they do to help the community when there is a disaster, shock, stressor or emergency. | [ ] With great difficulty [ ] Fairly easily  [ ] With difficulty [ ] Easily  [ ] With some difficulty [ ] Very easily |
| Probe | What does this mean to you? How would you put it in your own words?  Can you give me some examples? | If the participant does not understand, how was it described by the interviewer?  Was this explanation successful? [ YES / NO ] |
| Type of question failure | [ ] Issues with sequencing, length or sensitivity [ ] Problematic response options  [ ] Inappropriate vocabulary and long sentences [ ] Temporal and spatial confusion  [ ] Accessing different cognitive domains [ ] Failure to resonate with the respondent’s world  view and reality | |
| Notes for Revisions |  | |

| Item # | Statement: Inclusion | Answer Choices |
| --- | --- | --- |
| SO.9 | You and your household feel involved in community decisions that will affect your household, cluster, or community. | [ ] Strongly agree [ ] Disagree  [ ] Agree [ ] Strongly disagree  [ ] Neither agree or disagree |
| Probe | What does this mean to you? How would you put it in your own words?  What does ‘involvement’ in decision-making look like to you when we’re talking about community-level decisions? | If the participant does not understand, how was it described by the interviewer?  Was this explanation successful? [ YES / NO ] |
| Type of question failure | [ ] Issues with sequencing, length or sensitivity [ ] Problematic response options  [ ] Inappropriate vocabulary and long sentences [ ] Temporal and spatial confusion  [ ] Accessing different cognitive domains [ ] Failure to resonate with the respondent’s world  view and reality | |
| Notes for Revisions |  | |
| Item # | Statement: Social Cohesion | Answer Choices |
| SO.10 | people living here are willing to help their neighbor | [ ] Strongly agree [ ] Disagree  [ ] Agree [ ] Strongly disagree  [ ] Neither agree or disagree |
| Probe | What does this mean to you? How would you put it in your own words?  Do you think this was different before and after the COVID-19 pandemic?  Does this change depending on anything? On which neighbor needs help? On the type of help needed? | If the participant does not understand, how was it described by the interviewer?  Was this explanation successful? [ YES / NO ] |
| Type of question failure | [ ] Issues with sequencing, length or sensitivity [ ] Problematic response options  [ ] Inappropriate vocabulary and long sentences [ ] Temporal and spatial confusion  [ ] Accessing different cognitive domains [ ] Failure to resonate with the respondent’s world  view and reality | |
| Notes for Revisions |  | |

| Item # | Statement: Social Cohesion | Answer Choices |
| --- | --- | --- |
| SO.11 | most people living in this settlement can be trusted | [ ] Strongly agree [ ] Disagree  [ ] Agree [ ] Strongly disagree  [ ] Neither agree or disagree |
| Probe | What does this mean to you? How would you put it in your own words?  What does trust mean to you?  Does this depend on anything? Which people we’re talking about? What task or thing you’re trusting them with? | If the participant does not understand, how was it described by the interviewer?  Was this explanation successful? [ YES / NO ] |
| Type of question failure | [ ] Issues with sequencing, length or sensitivity [ ] Problematic response options  [ ] Inappropriate vocabulary and long sentences [ ] Temporal and spatial confusion  [ ] Accessing different cognitive domains [ ] Failure to resonate with the respondent’s world  view and reality | |
| Notes for Revisions |  | |
| Item # | Statement: Social Cohesion | Answer Choices |
| SO.12 | People in this generally do not get along with each other | [ ] Strongly agree [ ] Disagree  [ ] Agree [ ] Strongly disagree  [ ] Neither agree or disagree |
| Probe | What does this mean to you? How would you put it in your own words?  What does it mean to you to ‘get along’? | If the participant does not understand, how was it described by the interviewer?  Was this explanation successful? [ YES / NO ] |
| Type of question failure | [ ] Issues with sequencing, length or sensitivity [ ] Problematic response options  [ ] Inappropriate vocabulary and long sentences [ ] Temporal and spatial confusion  [ ] Accessing different cognitive domains [ ] Failure to resonate with the respondent’s world  view and reality | |
| Notes for Revisions |  | |

| Item # | Statement: Social Cohesion | Answer Choices |
| --- | --- | --- |
| SO.13 | People in this community would contribute money or labor to households that needed repairs after a distaster even if they were not family. | [ ] Strongly agree [ ] Disagree  [ ] Agree [ ] Strongly disagree  [ ] Neither agree or disagree |
| Probe | What does this mean to you? How would you put it in your own words?  What do you think of when I say ‘cost sharing’?  Does this depend on anything? Which household? How much the repairs costs? What the repairs are for exactly? | If the participant does not understand, how was it described by the interviewer?  Was this explanation successful? [ YES / NO ] |
| Type of question failure | [ ] Issues with sequencing, length or sensitivity [ ] Problematic response options  [ ] Inappropriate vocabulary and long sentences [ ] Temporal and spatial confusion  [ ] Accessing different cognitive domains [ ] Failure to resonate with the respondent’s world  view and reality | |
| Notes for Revisions |  | |
| Item # | Statement: Intrinsic (Self Efficacy) | Answer Choices |
| SO.14 | I feel confident in my ability to contribute to the community's efforts to overcome shocks/stressors | [ ] Strongly agree [ ] Disagree  [ ] Agree [ ] Strongly disagree  [ ] Neither agree or disagree |
| Probe | What does this mean to you? How would you put it in your own words?  What do you think of when I say pursuing common goals? | If the participant does not understand, how was it described by the interviewer?  Was this explanation successful? [ YES / NO ] |
| Type of question failure | [ ] Issues with sequencing, length or sensitivity [ ] Problematic response options  [ ] Inappropriate vocabulary and long sentences [ ] Temporal and spatial confusion  [ ] Accessing different cognitive domains [ ] Failure to resonate with the respondent’s world  view and reality | |
| Notes for Revisions |  | |

| Item # | Statement: Intrinsic (Collective Efficacy) | Answer Choices |
| --- | --- | --- |
| SO.15 | I feel confident that this community has the ability to successfully work together to overcome shocks/stressors | [ ] Strongly agree [ ] Disagree  [ ] Agree [ ] Strongly disagree  [ ] Neither agree or disagree |
| Probe | What does this mean to you? How would you put it in your own words? | If the participant does not understand, how was it described by the interviewer?  Was this explanation successful? [ YES / NO ] |
| Type of question failure | [ ] Issues with sequencing, length or sensitivity [ ] Problematic response options  [ ] Inappropriate vocabulary and long sentences [ ] Temporal and spatial confusion  [ ] Accessing different cognitive domains [ ] Failure to resonate with the respondent’s world  view and reality | |
| Notes for Revisions |  | |
| Item # | Statement: Collective Decision-making | Answer Choices |
| SO.16 | I can influence decisions about how the community will protect itself from or respond to shocks/stressors | [ ] With great difficulty [ ] Fairly easily  [ ] With difficulty [ ] Easily  [ ] With some difficulty [ ] Very easily |
| Probe | What does this mean to you? How would you put it in your own words? | If the participant does not understand, how was it described by the interviewer?  Was this explanation successful? [ YES / NO ] |
| Type of question failure | [ ] Issues with sequencing, length or sensitivity [ ] Problematic response options  [ ] Inappropriate vocabulary and long sentences [ ] Temporal and spatial confusion  [ ] Accessing different cognitive domains [ ] Failure to resonate with the respondent’s world  view and reality | |
| Notes for Revisions | Drop for scale; keep for CI | |

| Item # | Statement: Collective Decision-making | Answer Choices |
| --- | --- | --- |
| SO.17 | Anyone in the community who wants to can be involved in deicison-making about how the community will protect itself from or respond to shocks/stressors | [ ] Strongly agree [ ] Disagree  [ ] Agree [ ] Strongly disagree  [ ] Neither agree or disagree |
| Probe | What does this mean to you? How would you put it in your own words? | If the participant does not understand, how was it described by the interviewer?  Was this explanation successful? [ YES / NO ] |
| Type of question failure | [ ] Issues with sequencing, length or sensitivity [ ] Problematic response options  [ ] Inappropriate vocabulary and long sentences [ ] Temporal and spatial confusion  [ ] Accessing different cognitive domains [ ] Failure to resonate with the respondent’s world  view and reality | |
| Notes for Revisions |  | |
| Item # | Statement:Collective Action | Answer Choices |
| SO.18 | I or members of my household usually contribute to community efforts to protect from or respond to shocks/stressors | [ ] With great difficulty [ ] Fairly easily  [ ] With difficulty [ ] Easily  [ ] With some difficulty [ ] Very easily |
| Probe | What does this mean to you? How would you put it in your own words? | If the participant does not understand, how was it described by the interviewer?  Was this explanation successful? [ YES / NO ] |
| Type of question failure | [ ] Issues with sequencing, length or sensitivity [ ] Problematic response options  [ ] Inappropriate vocabulary and long sentences [ ] Temporal and spatial confusion  [ ] Accessing different cognitive domains [ ] Failure to resonate with the respondent’s world  view and reality | |
| Notes for Revisions | Drop for scale; keep for CI | |

| Item # | Statement: Collective Action | Answer Choices |
| --- | --- | --- |
| SO.19 | It is normal for people in this community to work together (meaning NOT every household for themselves) to protect from or respond to shocks/stressors. | [ ] Strongly agree [ ] Disagree  [ ] Agree [ ] Strongly disagree  [ ] Neither agree or disagree |
| Probe | What does this mean to you? How would you put it in your own words? | If the participant does not understand, how was it described by the interviewer?  Was this explanation successful? [ YES / NO ] |
| Type of question failure | [ ] Issues with sequencing, length or sensitivity [ ] Problematic response options  [ ] Inappropriate vocabulary and long sentences [ ] Temporal and spatial confusion  [ ] Accessing different cognitive domains [ ] Failure to resonate with the respondent’s world  view and reality | |
| Notes for Revisions |  | |
| Item # | Statement: Collective Action | Answer Choices |
| SO.20 | People in this community are motivated to overcome shocks/stressors, even when it seems challenging to do so | [ ] Strongly agree [ ] Disagree  [ ] Agree [ ] Strongly disagree  [ ] Neither agree or disagree |
| Probe | What does this mean to you? How would you put it in your own words? | If the participant does not understand, how was it described by the interviewer?  Was this explanation successful? [ YES / NO ] |
| Type of question failure | [ ] Issues with sequencing, length or sensitivity [ ] Problematic response options  [ ] Inappropriate vocabulary and long sentences [ ] Temporal and spatial confusion  [ ] Accessing different cognitive domains [ ] Failure to resonate with the respondent’s world  view and reality | |
| Notes for Revisions | Drop for scale; keep for CI | |

| Item # | Statement: Common Good | Answer Choices |
| --- | --- | --- |
| SO.21 | Working together/doing communal work to protect the community from shocks/stressors before they happen is a good use of our time and/or money. This might include giving money, labor, or land for digging drainage ditches or building sea walls. | [ ] Strongly agree [ ] Disagree  [ ] Agree [ ] Strongly disagree  [ ] Neither agree or disagree |
| Probe | What does this mean to you? How would you put it in your own words? | If the participant does not understand, how was it described by the interviewer?  Was this explanation successful? [ YES / NO ] |
| Type of question failure | [ ] Issues with sequencing, length or sensitivity [ ] Problematic response options  [ ] Inappropriate vocabulary and long sentences [ ] Temporal and spatial confusion  [ ] Accessing different cognitive domains [ ] Failure to resonate with the respondent’s world  view and reality | |
| Notes for Revisions | Drop for scale; keep for CI | |
| Item # | Statement: Common Good | Answer Choices |
| SO.22 | Working together/doing communal work to clean up or rebuild the community after shocks/stressors is a good use of our time and/or money. This might include community clean ups to clear debris or giving money or labor to rebuild communal structures. | [ ] Strongly agree [ ] Disagree  [ ] Agree [ ] Strongly disagree  [ ] Neither agree or disagree |
| Probe | What does this mean to you? How would you put it in your own words?  Can you talk about your last time taking part in a clean-up activity or rebuilding the community? | If the participant does not understand, how was it described by the interviewer?  Was this explanation successful? [ YES / NO ] |
| Type of question failure | [ ] Issues with sequencing, length or sensitivity [ ] Problematic response options  [ ] Inappropriate vocabulary and long sentences [ ] Temporal and spatial confusion  [ ] Accessing different cognitive domains [ ] Failure to resonate with the respondent’s world  view and reality | |
| Notes for Revisions | Drop for scale; keep for CI | |

| Section 3.3 Social Resilience: Learning and Innovation | | |
| --- | --- | --- |
| Item # | Statement: Information | Answer Choices |
| SO.23 | Your household receives information about potential or predicted storms, floods, disasters, droughts etc., with enough time to plan and make decisions for the safety of your household and/or livelihoods. | [ ] Strongly agree [ ] Disagree  [ ] Agree [ ] Strongly disagree  [ ] Neither agree or disagree |
| Probe | What does this mean to you? How would you put it in your own words? | If the participant does not understand, how was it described by the interviewer?  Was this explanation successful? [ YES / NO ] |
| Type of question failure | [ ] Issues with sequencing, length or sensitivity [ ] Problematic response options  [ ] Inappropriate vocabulary and long sentences [ ] Temporal and spatial confusion  [ ] Accessing different cognitive domains [ ] Failure to resonate with the respondent’s world  view and reality | |
| Notes for Revisions |  | |
| Item # | Statement: Information | Answer Choices |
| SO.24 | You or someone in your household has knowledge or training on what to do before, during, and after a natural disaster or weather related hazard. | [ ] Strongly agree [ ] Disagree  [ ] Agree [ ] Strongly disagree  [ ] Neither agree or disagree |
| Probe | What does this mean to you? How would you put it in your own words? | If the participant does not understand, how was it described by the interviewer?  Was this explanation successful? [ YES / NO ] |
| Type of question failure | [ ] Issues with sequencing, length or sensitivity [ ] Problematic response options  [ ] Inappropriate vocabulary and long sentences [ ] Temporal and spatial confusion  [ ] Accessing different cognitive domains [ ] Failure to resonate with the respondent’s world  view and reality | |
| Notes for Revisions |  | |

| Item # | Statement: Information | Answer Choices |
| --- | --- | --- |
| SO.25 | I would know where to go to get information on how to protect myself and my household from environmental shocks and stressors such as typhoons, floods, droughts, or disease outbreaks. | [ ] Strongly agree [ ] Disagree  [ ] Agree [ ] Strongly disagree  [ ] Neither agree or disagree |
| Probe | What does this mean to you? How would you put it in your own words? | If the participant does not understand, how was it described by the interviewer?  Was this explanation successful? [ YES / NO ] |
| Type of question failure | [ ] Issues with sequencing, length or sensitivity [ ] Problematic response options  [ ] Inappropriate vocabulary and long sentences [ ] Temporal and spatial confusion  [ ] Accessing different cognitive domains [ ] Failure to resonate with the respondent’s world  view and reality | |
| Notes for Revisions |  | |
| Item # | Statement: Mitigation | Answer Choices |
| SO.26 | Planning and preparing for natural disasters and weather related hazards is a priority for my household. | [ ] Strongly agree [ ] Disagree  [ ] Agree [ ] Strongly disagree  [ ] Neither agree or disagree |
| Probe | What does this mean to you? How would you put it in your own words? | If the participant does not understand, how was it described by the interviewer?  Was this explanation successful? [ YES / NO ] |
| Type of question failure | [ ] Issues with sequencing, length or sensitivity [ ] Problematic response options  [ ] Inappropriate vocabulary and long sentences [ ] Temporal and spatial confusion  [ ] Accessing different cognitive domains [ ] Failure to resonate with the respondent’s world  view and reality | |
| Notes for Revisions |  | |

Final Probes:

1. Is there anything else you want us to know about your experiences related to these questions?

2. Overall, what did you think of this survey?

3. Were the questions difficult or easy to understand? Why?

4. Do you have any suggestions or recommendations for improving the survey?
